# Synaptotagmin-7 deficit causes insulin hypoactivity and contributes to behavioral alterations in mice

**DOI:** 10.1016/j.isci.2025.112354

**Published:** 2025-04-04

**Authors:** Yao-Nan Liu, Qiu-Wen Wang, Si-Yao Lu, Wei Shen, Chongye Guo, Zhikai Xing, Chang Li, Shan Sun, Sen-Fang Sui, Shuangli Mi, Fred H. Gage, Jun Yao

**Affiliations:** 1 State Key Laboratory of Membrane Biology, IDG/McGovern Institute for Brain Research, School of Life Sciences, Tsinghua University, Beijing 100084, China; 2 Laboratory of Genetics, The Salk Institute for Biological Studies, La Jolla, CA 92037, USA; 3 Jiangsu Key Laboratory of Language and Cognitive Neuroscience, School of Linguistic Sciences and Arts, Jiangsu Normal University, Xuzhou 221116, China; 4 Jiangsu Collaborative Innovation Center for Language Ability, Xuzhou 221009, China; 5 China National Center for Bioinformation, Beijing Institute of Genomics, Chinese Academy of Sciences, Beijing 100101, China; 6 University of Chinese Academy of Sciences, Beijing 100049, China; 7 State Key Laboratory of Membrane Biology, Beijing Frontier Research Center for Biological Structure, School of Life Sciences, Tsinghua University, Beijing 100084, China

**Keywords:** Natural sciences, biological sciences, neuroscience, behavioral neuroscience, molecular neuroscience

## Abstract

Synaptotagmin-7 (Syt7) KO mice show diurnal fluctuations of mania- and depression-like behavioral abnormalities. Although GluN2B-NMDAR hypoactivity has been shown to be involved in the induction of mania-like behaviors of the Syt7 KO mice in the dark phase, the reasons for the depression-like behaviors in the light phase and behavioral fluctuation remain unknown. Here, we show that bipolar I disorder (BDI)-patient-induced pluripotent stem cell (iPSC)-derived islet-like organoids exhibited Syt7-dependent insulin secretion defects; moreover, Syt7-deficiency-induced insulin hyposecretion generated depression-like behaviors in Syt7 KO mice in the light phase. Furthermore, pancreatic insulin secretion and neuronal activity showed opposite diurnal patterns, in which the Syt7-deficiency-induced disequilibrium induced periodic antagonistic shifts in the mania- and depression-like behaviors. Finally, using RNA sequencing (RNA-seq) analysis, we explored downstream pathways that might underlie the diurnal fluctuation of behaviors. Therefore, Syt7-deficiency-induced insulin hypoactivity contributed to light-phase depression-like behaviors and diurnal behavioral fluctuations in the mice.

## Highlights

• Syt7-deficiency-induced GSIS deficits generate depression-like behaviors
• Islet insulin secretion and hippocampal activity have distinct diurnal patterns
• Islet/hippocampal diurnal rhythm discrepancy induces behavioral fluctuations
• BDI patient iPSC-derived islet-like organoids show insulin and Syt7 deficits

## Graphical abstract

**Figure:**
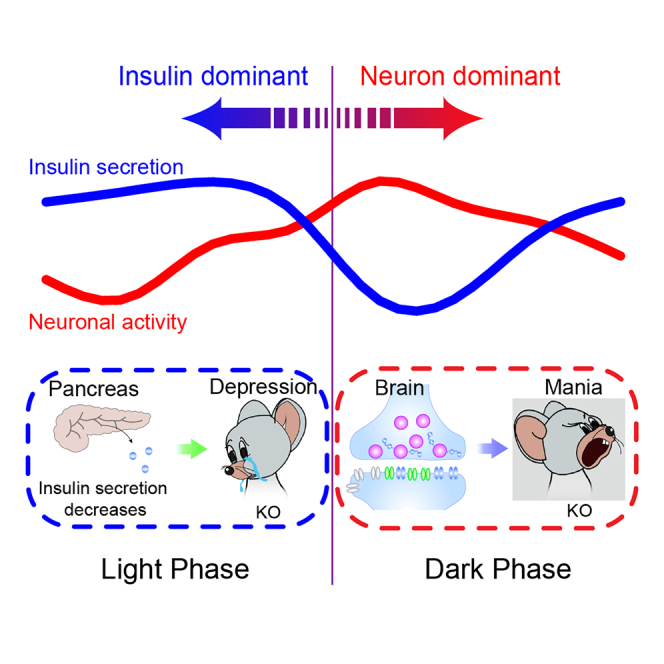

## Introduction

Bipolar disorder (BD) is characterized by complex intermittent episodes of mania and depression. As pedigree analysis has suggested a strong heritability of this mental illness, dysfunction of some molecular pathway may not be only crucial for the behavioral abnormalities but can also be stably inherited, and diverse or multiple susceptible genes of BD might functionally converge onto this critical molecular pathway.^1^ Accompanied by mood cycling, BD patients often show cardiovascular or metabolic comorbidities. One such example is type 2 diabetes (T2D), which shows a prevalence of >10% within the BD population and significantly exacerbates the outcome of BD.^2^ Even in BD patients without detectable T2D, more than one-third suffer from pre-diabetes or high blood glucose, indicating that BD patients generally harbor a high risk for insulin metabolic abnormalities.^3^^,^^4^^,^^5^ In clinical practice, insulinopenia has often been observed in the brains of BD patients^6^^,^^7^; moreover, several studies have suggested that insulin sensitizers such as pioglitazone showed promising efficacy in the treatment of bipolar depression patients with or without metabolic syndrome.^3^^,^^8^^,^^9^^,^^10^^,^^11^^,^^12^ Therefore, the molecular mechanism critical for behavioral symptoms might also contribute to the induction of metabolic abnormalities in the periphery.

Synaptotagmin-7 (Syt7) plays important roles in synaptic transmission in the brain and insulin secretion in the pancreas. Syt7 drives the exocytosis of secretory vesicles through its two tandem Ca^2+^-binding domains interacting with Ca^2+^, SNAREs, and phospholipids. In the brain, Syt7 deficiency causes an attenuation in action potential (AP)-triggered asynchronous neurotransmitter release, synaptic vesicle replenishment and synaptic facilitation in the presynaptic nerve terminals, as well as AMPAR trafficking in the postsynaptic density.^13^^,^^14^^,^^15^^,^^16^^,^^17^ Previously, it has been suggested that, in pancreatic β-cells, Syt7 plays an essential role in promoting glucose-stimulated insulin secretion (GSIS).^18^^,^^19^ The glucagon-like peptide-1 (GLP-1) pathway, one therapeutic target of T2D drugs, can upregulate the phosphorylation of Syt7 to facilitate insulin secretion.^20^ Hence, Syt7 may function in both the neural and metabolic disorders. Consistently, we have observed that Syt7 knockout (KO) mice can show mania-like behaviors in the dark phase and depression-like behaviors in the light phase^1^ and demonstrated that Syt7 can regulate manic-like behaviors through modulating the activation of GluN2B-containing NMDARs (GluN2B-NMDARs) in the peripheral synaptic region.^21^ However, the mechanisms underlying the induction of depression-like behaviors and behavioral fluctuations in the Syt7 KO mice remain unclear.

In the present study, we show that in Syt7 KO mice, insulin secretion deficits induced depression-like behaviors in the light phase directly through insulin receptors in the brain. The pancreatic insulin secretion and hippocampal neuronal activity, which has been shown to be associated with mania-like behaviors through activation of GluN2B-NMDARs, showed opposite diurnal patterns. Thus, Syt7 deficiency induced a periodic antagonistic shift in the balance of pancreatic and brain activities, resulting in a behavioral fluctuation phenotype. Further, we performed RNA sequencing (RNA-seq) analysis in the hippocampus of mice and observed that autoimmune pathways involved in the comorbidities of BD might function downstream to insulin to contribute to the behavioral fluctuations in mice. Finally, we showed that the BD patient induced pluripotent stem cell (iPSC)-derived islet-like organoids had Syt7-dependent insulin secretion deficits. Together, our results might contribute to understanding the induction of bipolar-like behavioral abnormalities in the Syt7 KO mice.

## Results

### Syt7-deficiency-induced GSIS deficits generate depression-like behaviors

**Figure 1:**
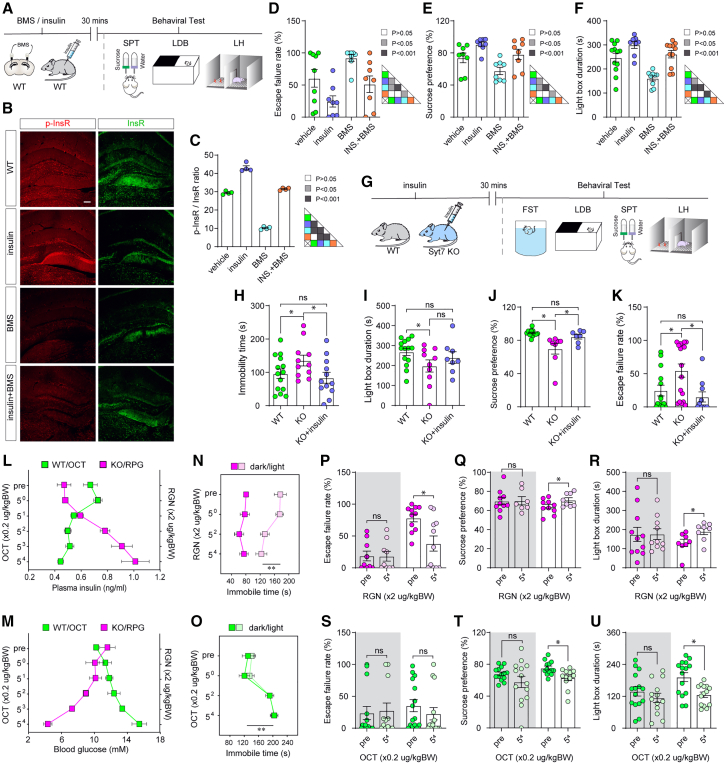
Syt7-deficiency-induced insulin secretion deficits cause depression-like behaviors (A) Schematic showing the intraperitoneal injection of insulin and brain infusion of BMS-536924 (BMS) and experimental tests in wild-type (WT) mice for (B–F). (B and C) Immunostaining images (B) and quantitative analysis of fluorescence intensity (C; *n* = 4) showing the regulation of InsR (insulin receptor) and p-InsR (phosphorylated insulin receptor) in the hippocampus by insulin/BMS. Scale bar, 100 μm. (D–F) Effects of peripheral insulin and brain BMS application on the LH (learned helplessness) escape failure rate (D; *n* = 9 [vehicle], 8 [insulin], 8 [BMS], 8 [insulin+BMS]), sucrose preference ratio (E; *n* = 8), and LDB (light/dark box) time-in-light (F. n = 12/8/9/11) of WT mice. (G) Schematic showing the intraperitoneal injection of insulin and experimental tests for (H–K). (H–K) Bar graphs summarizing the FST (forced swim test) immobility time (H; *n* = 14 [WT], 11 [Syt7 KO], 12 [Syt7 KO + insulin]), LDB time-in-light (I; *n* = 14/10/8), sucrose preference ratio (J; *n* = 13/8/7), and LH escape failure rate (K; *n* = 14/17/10) following intraperitoneal injection of insulin into Syt7 KO mice. (L and M) Graphs showing the plasma insulin (L) or blood glucose (M) concentration in WT mice receiving the octreotide (OCT) treatment and in Syt7 KO mice receiving the repaglinide (RGN) treatment. *n* = 8. (N and O) Graphs summarizing FST immobility time of Syt7 KO mice treated with RGN (N) or WT mice receiving OCT treatment (O). FST analysis was performed in Syt7 KO mice treated with varying RGN doses (N) and WT mice treated with gradient OCT concentrations (O) during both the light and dark phases. The experimental results indicated that RGN reduced the immobility time of the Syt7 KO mice at high doses in the light phase but did not induce any significant effects in the dark phase, whereas OCT increased the immobility time of the WT KO mice at high doses in both the dark and light phases. *n* = 8. (P–R) Graphs summarizing the LH escape failure rate (P; dark, *n* = 9 [Syt7 KO], 9 [Syt7 KO + RGN]; light, *n* = 11 [Syt7 KO], 11 [Syt7 KO + RGN]), sucrose preference ratio (Q; dark, *n* = 10/7; light, *n* = 10/8), and LDB time-in-light (R; dark phase, *n* = 11/10; light phase, *n* = 8/8) of Syt7 KO mice treated with high-dose RGN. During the light phase, RGN treatment induced a decreased LH escape failure rate, an increased sucrose preference ratio, and an increased LDB time-in-light in the Syt7 KO mice. In contrast, during the dark phase, RGN had only slight effects. (S–U) Graphs summarizing the LH escape failure rate (S; dark, *n* = 14 [WT], 12 [WT + OCT]; light, *n* = 15 [WT], 14 [WT + OCT], sucrose preference ratio (T; *n* = 14), and LDB time-in-light (U; dark, *n* = 15/13; light, *n* = 15/14) of WT mice treated with OCT. During the light phase, OCT treatment reduced the sucrose preference ratio and the LDB time-in-light of the WT mice, whereas the LH escape failure rate remained unchanged. One-way ANOVA with Sidak’s multiple comparisons test; ∗*p* < 0.05; ∗∗*p* < 0.001; error bars, SEM.

Previously, it has been suggested that anti-diabetic drugs such as pioglitazone can be used to treat bipolar depression.^3^^,^^8^^,^^9^^,^^10^^,^^11^^,^^12^ We investigated the direct effects of insulin release on animal behaviors in mice. We treated wild-type (WT) mice with insulin through intraperitoneal injection at ZT 4-6 and performed behavioral tests, including learned helplessness (LH), light/dark box (LDB), and sucrose preference tests (SPT), after 30 min (Figure 1A). The LH and SPT were often used to indicate the extent of stress and anhedonia, respectively, and the LDB test was used to investigate the anxiety-like behaviors of the mice. As insulin receptors (InsRs) are highly enriched in the dentate gyrus (DG),^22^ we infused BMS-536924, an insulin receptor (InsR) blocker, into the DG of the hippocampus to assay the effects of brain InsRs on mouse behaviors. Immunostaining analysis revealed that intraperitoneal insulin application upregulated the InsR phosphorylation (p-InsR) level in the hippocampus, whereas the pre-infusion of BMS-536924 before insulin application could partially antagonize the insulin-induced enhancement of InsR phosphorylation (Figures 1B and 1C). The results of behavioral analyses showed that insulin application significantly reduced the LH escape failure rate and increased the sucrose preference ratio and LDB time-in-light, whereas the BMS-536924 treatment induced opposite outcomes (Figures 1D–1F).

Previously, we found that the Syt7 KO mice could show mania-like behaviors in the dark phase (Figure S1), whereas in the light phase, the Syt7 KO mice showed depression-like behaviors.^1^^,^^21^ Here, we performed multiple paradigms, including the LH, LDB, SPT, and forced swim test (FST), to verify our observations in the light phase. The FST tests were often used to assay the response to acute stress of the animals. The results indicated that, compared to the WT controls, the Syt7 KO mice showed an increased LH escape failure rate, decreased sucrose preference ratio and LDB time-in-light, and increased FST immobility time (Figures 1G–1K). We treated the Syt7 KO mice with insulin in the light phase and observed that the insulin treatment alleviated the behavioral abnormalities of the KO mice.

It has been suggested that insulin is secreted not only from pancreatic β-cells but also in some brain regions, including the hippocampus.^23^^,^^24^ Hence, we investigated the co-localization of Syt7 and insulin in multiple regions in the mouse brain and observed that in all tested brain regions, Syt7 and insulin showed sparse co-localization (Figure S2). Further, we used cultured hippocampal neurons to test whether Syt7 could affect brain insulin secretion. In the cultured neurons, Syt7 was not co-localized with insulin (Figure S3A), consistent with our observations in the mouse brain. Moreover, the GSIS measurement with an enzyme-linked immunosorbent assay (ELISA) did not detect a significant difference between the WT and Syt7 KO neurons (Figure S3B). In contrast to the cultured neurons, Syt7 and insulin were highly co-localized in acutely dissected pancreatic β-cells (Figure S3C). We assayed the pancreatic GSIS of the mice by measuring the changes in plasma insulin caused by intraperitoneal injection of glucose. Consistent with previous research,^18^ the GSIS in the diurnal period was largely abolished in the Syt7 KO mice (Figure S3D). As GSIS is crucial for maintaining insulin homeostasis, the baseline plasma insulin concentration in the Syt7 KO mice was remarkably lower than in the WT animals (Figure S3E). These results indicated that Syt7 was involved in depression-like behaviors by regulating insulin release in pancreatic islets but not in the hippocampus.

We were then interested to know whether clinical insulin-modulating drugs can be used to alleviate the behavioral abnormalities shown by the Syt7 KO mice. To this end, we administered repaglinide (RGN), a clinical anti-diabetic drug, to the Syt7 KO mice to promote insulin secretion and decrease the blood glucose level (Figures 1L and 1M). FST analysis in Syt7 KO mice treated with varying doses of repaglinide indicated that high-dose repaglinide could significantly decrease the FST immobility time of the KO mice in the light phase but not in the dark phase (Figure 1N). Further behavioral tests in the Syt7 KO mice with high-dose repaglinide treatment revealed that, in the light phase, the treatment of repaglinide induced decreased LH escape failure rate and increased sucrose preference ratio and LDB time-in-light; in contrast, in the dark phase, repaglinide only had slight effects (Figures 1P–1R). These results indicated that the light-phase behavioral deficits of the Syt7 KO mice were induced by insulin deficits and could be alleviated by repaglinide.

In addition, we treated the WT mice with octreotide (OCT), a mimic of somatostatin that can attenuate insulin secretion and elevate the blood glucose level (Figures 1L and 1M). FST analysis in WT mice treated with gradient concentrations of octreotide indicated that high-dose octreotide significantly increased the FST immobility time in both the dark and light phases (Figure 1O). However, further behavioral tests with high-dose octreotide treatment showed that in the light phases, octreotide could decrease the sucrose preference ratio and LDB time-in-light while the LH escape failure rate was unaffected; in contrast, in the dark phase, octreotide failed to significantly affect the behaviors of the mice (Figures 1S–1U).

Together, our results indicated that the GSIS deficits in the Syt7 KO mice could induce depression-like behavioral abnormalities in the light phase, which were alleviated by the treatment of anti-diabetic drugs that stimulate insulin secretion.

### Distinct diurnal patterns of pancreatic insulin secretion and hippocampal neuronal spiking contribute to behavioral fluctuations

Previously, we demonstrated that, in the hippocampus, Syt7 triggered glutamate release in the peripheral presynaptic region to efficiently activate the juxtaposed postsynaptic GluN2B-containing NMDARs.^21^ Deficits in this process directly induced antidepressant effects in Syt7-deficient mice because Ro25-6981, an antagonist specific for GluN2B-NMDARs, could induce significant antidepressant effects on the WT mice but had moderate effects on the Syt7 KO mice (Figure S1). Here, we asked how the insulin-deficit-induced depression-like behaviors and the hippocampal-NMDAR-hypofunction-induced mania-like behaviors appeared alternately instead of canceling each other out. As the behavioral fluctuations of the Syt7 KO mice exhibited a diurnal changing pattern, we assayed the 24-h rhythms of the functions of Syt7 in the brain and pancreas.

**Figure 2:**
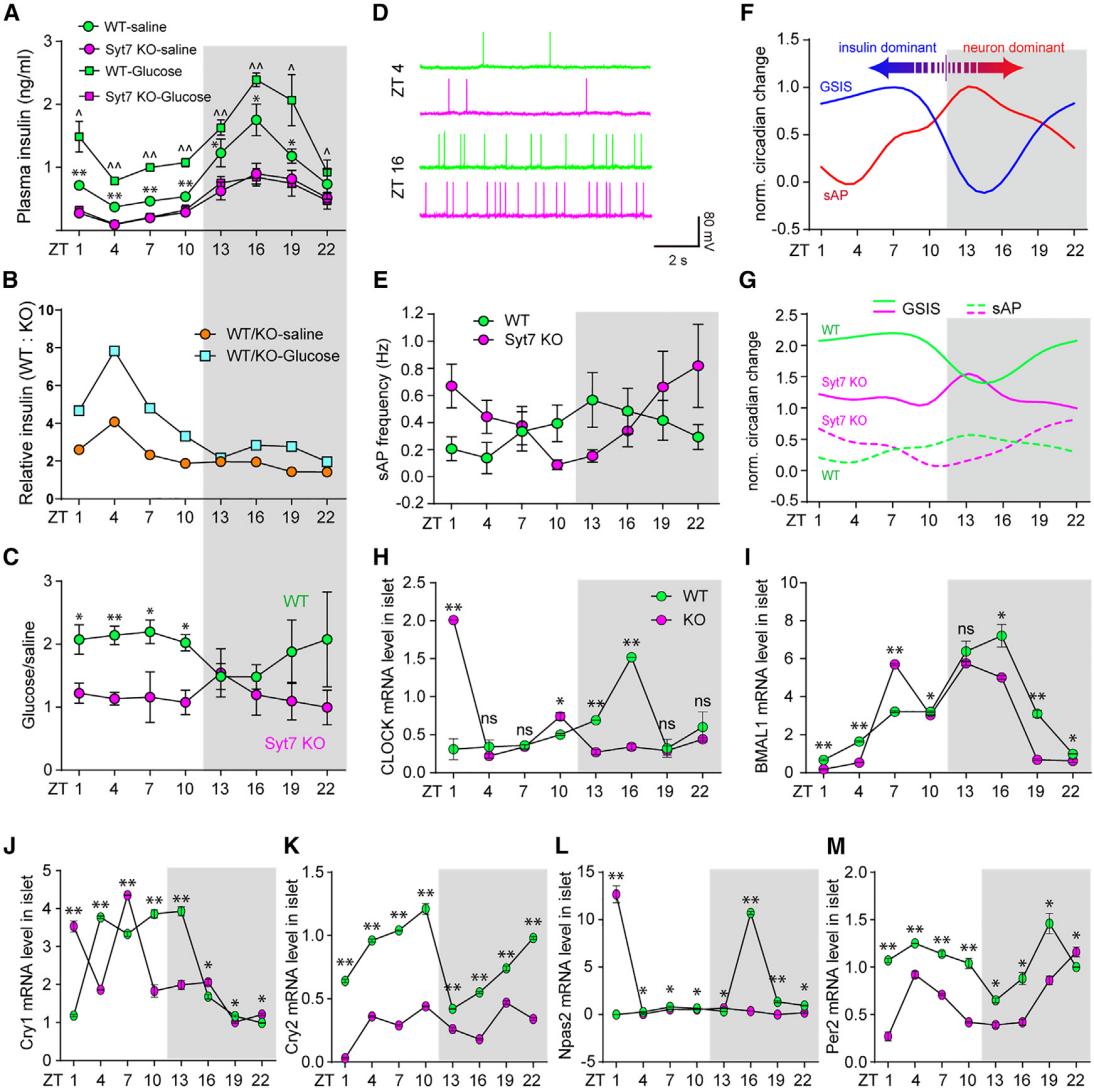
Distinct circadian rhythms of insulin secretion and hippocampal neuronal activity (A–C) Diurnal plasma insulin changing patterns of wild-type (WT) and Syt7 KO mice receiving glucose stimulation or saline treatment. *n* = 12 (WT), 8 (KO). (A) Diurnal changing pattern of plasma insulin concentration. (B) Diurnal changes of the ratio of resting or post-stimulation insulin concentration in WT mice to that in Syt7 KO mice. (C) Diurnal changing pattern of glucose-stimulated insulin secretion (GSIS) in WT and Syt7 KO mice. (D and E) Representative traces (D) and quantitative analysis (E) showing a diurnal changing pattern of spontaneous action potential (sAP) firing frequency in hippocampal dentate gyrus (DG) slices. *n* = 20∼33 neurons of six mice. (F) Normalized diurnal patterns of hippocampal sAP firing and pancreatic GSIS in WT mice. (G) Comparison of diurnal patterns of hippocampal sAP firing frequency and pancreatic GSIS in WT and Syt7 KO mice. Syt7-deficiency-induced loss of sAP firing and GSIS domination in the dark and light phases. (H–M) The 24-h diurnal expressing pattern of circadian genes [including CLOCK (H), BMAL1 (I), Cry1 (J), Cry2 (K), Npas2 (L), and Per2 (M)] in the islet of the WT and Syt7 KO mice. *n* = 3. Cry2, cryptochrome circadian regulator 2; Per2, period circadian regulator 2; CLOCK, clock circadian regulator; Npas2, neuronal PAS domain protein 2; BMAL1, basic helix-loop-helix ARNT-like 1; Cry1, cryptochrome circadian regulator 1. (A–M) two-way ANOVA with Sidak’s multiple comparison test; ∗*p* < 0.05; ∗∗*p* < 0.001; error bars, SEM.

We first measured the 24-h rhythms of the baseline plasma insulin level and GSIS. Throughout the 24 h, the Syt7 KO mice showed persistently lower baseline and stimulated insulin levels than the WT mice (Figure 2A). In both groups, the baseline and post-stimulation insulin showed an obvious circadian rhythm, reaching a peak in the dark phase and a nadir in the light phase. However, the Syt7-KO-induced attenuation in the baseline and stimulated insulin levels, depicted by the fold change of insulin level in the WT mice over the Syt7 KO mice, had an opposite changing pattern, maximized in the light phase and minimized in the dark phase (Figure 2B). Based on the baseline and post-stimulation insulin level data, we analyzed the GSIS of the WT and Syt7 KO mice, which was determined by the ratio of glucose-stimulated versus baseline insulin concentration. The results indicated that, in the WT mice, the GSIS also exhibited a diurnal rhythm maximized in the light phase and minimized in the dark period, exhibiting an approximately 2-fold difference, whereas in the Syt7 KO mice, the GSIS failed to show any obvious diurnal changing pattern (Figure 2C). Hence, the Syt7-deficiency-induced insulin deficits were much more drastic in the light phase than in the dark phase (Figures 2A–2C). We next assayed the circadian rhythm of the spontaneous AP firing in the hippocampus, which triggered glutamate release to activate GluN2B-NMDARs that were crucial for the induction of mania-like behaviors. To this end, we performed patch-clamp recordings in hippocampal DG slices of the WT mice on a 24-h scale with an interval of 3 h (Figure 2D). The AP firing frequency exhibited a diurnal cycling pattern with a peak in the dark phase and a nadir in the light phase, showing an approximately 4-fold difference; however, in the Syt7 KO mice, the diurnal AP firing pattern was changed (Figure 2E). These results indicated that in the WT mice, the pancreatic GSIS and hippocampal neuronal activity showed opposite diurnal fluctuating patterns (Figure 2F). Therefore, we hypothesized that, in the Syt7 KO mice, deficits in AP-dependent GluN2B-NMDAR activity dominated the behaviors of the animals in the dark period, resulting in a mania-like phenotype, whereas in the light period, defects in the GSIS became the leading factor compelling the animals into a depression-like state (Figure 2G).

To test the long-term influence of persistent insulin hyposecretion in the Syt7 KO mice, we measured the 24-h expressing patterns of circadian genes in the islets of these animals. In the light phase, compared to the WT group, Cry2 (cryptochrome circadian regulator 2) and Per2 (period circadian regulator 2) were decreased, CLOCK (clock circadian regulator) and Npas2 (neuronal PAS domain protein 2) were increased, and BMAL1 (basic helix-loop-helix ARNT-like 1) and Cry1 (cryptochrome circadian regulator 1) were slightly affected (Figures 2G–2L). In the dark phase, CLOCK, BMAL1, Cry1, Cry2, Npas2, and Per2 were decreased. In addition, we found that the 24-h expressing patterns of circadian genes in the hippocampus of the Syt7 KO mice were also changed (Figures S4A–S4F).

Together, our results indicate that the Syt7-triggered pancreatic GSIS and hippocampal GluN2B activation have opposite diurnal rhythms, which might be a reason for the diurnal fluctuation of behaviors in the Syt7 KO mice.

### Signaling pathways downstream to GluN2B and insulin in mood cycling

**Figure 3:**
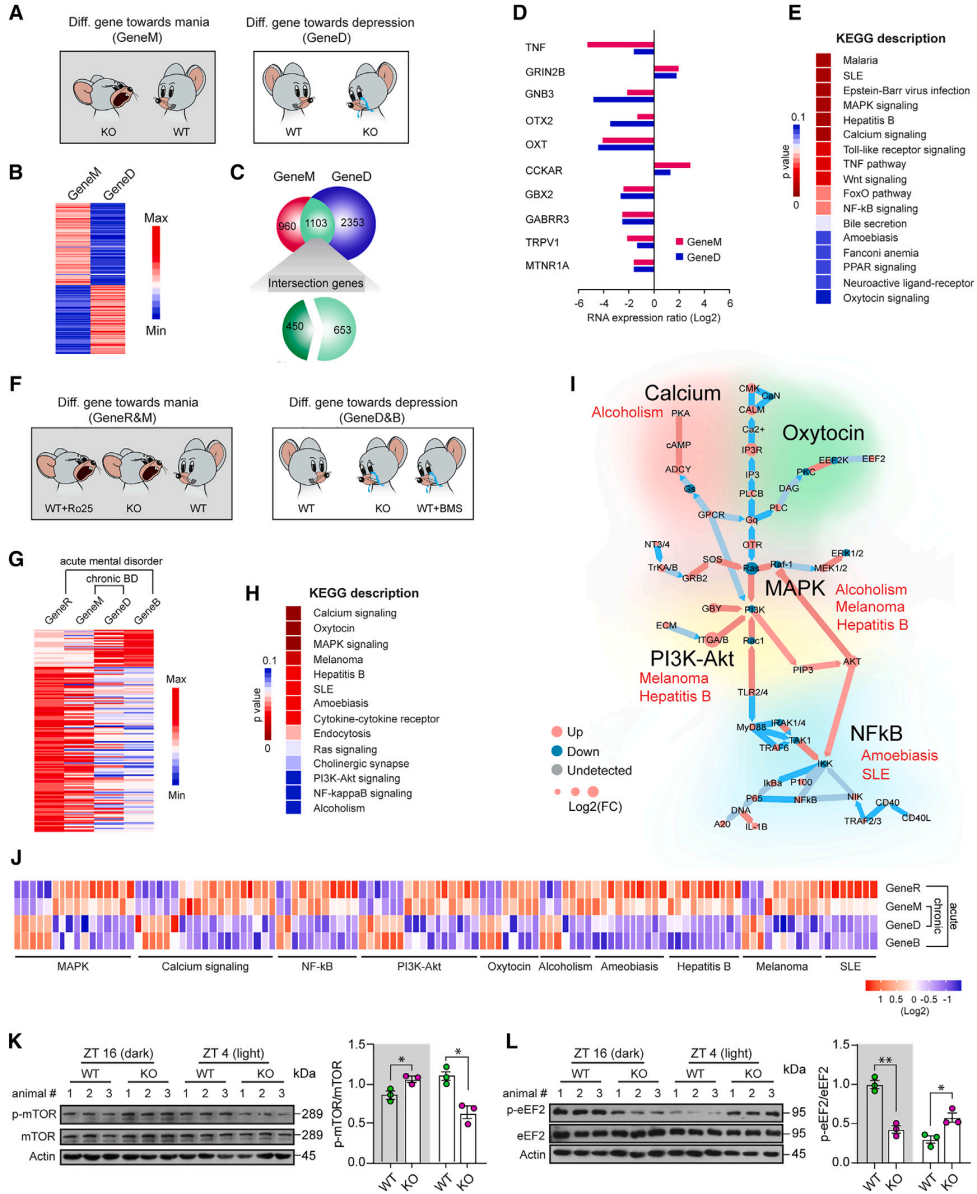
Signaling pathways involved in the genesis of mood cycling (A) Schematic showing comparative analysis of hippocampal gene expression profiles between manic and depressive Syt7 KO mice. GeneM, comparison between the WT mice and the manic Syt7 KO mice. GeneD, comparison between the WT mice and the depressive Syt7 KO mice. (B and C) Heatmap (B) and distribution (C) of genes differentially expressed between GeneM and GeneD. (D) Expression of representative BD-related genes in manic and depressive Syt7 KO mice. (E) Representative significantly enriched KEGG pathways showing bi-directional alterations in the manic and depressive episodes in Syt7 KO mice. (F and G) Schematic (F) and heatmap (G) showing the comparative analysis of transcripts bi-directionally regulated in drug-induced/Syt7 deficiency-induced manic and depressive mice. GeneR, comparison between the WT mice with and without the Ro25 treatment in the dark phase. GeneB, comparison between the WT mice with and without the BMS-536924 treatment in the light phase. (H) Representative significantly enriched KEGG pathways showing bi-directional alterations between the drug-induced/Syt7-deficiency-induced manic and depressive mice. (I) Mapping of representative KEGG pathways for network analysis. (J) Bi-directional regulation of representative genes within featured KEGG pathways in mice with drug-induced/Syt7-deficiency-induced mania or depression. (K and L) Immunoblots (left) and quantitative analysis (right) of mammalian targets of rapamycin (mTOR) (K) and eukaryotic elongation factor 2 (eEF2) (L) phosphorylation in the hippocampus of WT and Syt7 KO mice. *n* = 3. Student’s t test; ∗*p* < 0.05; ∗∗*p* < 0.001; error bars, SEM.

To explore the *in vivo* signaling pathways downstream to the insulin involved in behavioral abnormalities. We performed RNA-seq analysis in the hippocampal tissues of Syt7 KO mice (Figures 3A–3C and S5). Our analysis revealed that those 1,103 genes were differentially expressed in both the dark and light phases. Many genes that showed genetic variants or expression changes in BD patients, including *GRIN2B* (encoding GluN2B), exhibited identical regulatory patterns in the Syt7 KO mice during the dark and light periods (Figure 3D). Further KEGG analysis revealed that 68 signaling pathways (*p* ≤ 0.05) in the hippocampus of the Syt7 KO mice showed bi-directional changes in the dark and light phases (Table S1), many of which are either featured in affective disorders or related to BD comorbidities such as systemic lupus erythematosus (SLE) (Figure 3E).

To distinguish the pathways altered directly by the Syt7 signaling from those adjusted developmentally, we employed two WT animal groups that were treated with BMS-536924 to directly induce depression-like behavioral abnormalities or with Ro25-6981, an antagonist specific for GluN2B-NMDARs, to induce manic-like behaviors (Figures 3F and 3G). Comparative analysis revealed 30 KEGG pathways that were bi-directionally regulated between mice showing mania-like and depression-like behaviors, of which 20 showed a significance of *p* ≤ 0.05 (Table S2). The mTOR-related PI3K-Akt pathway showed low significance. Because we observed a noticeable fluctuation in the mTOR activity during behavioral fluctuations (Figure 3L), we included all 30 pathways to avoid the loss of any important one (Figure 3H). These pathways were grouped into three categories: (1) pathways identified in BD patients and drug pharmacology, including the Ca^2+^ signaling, oxytocin, MAPK, NF-κB, Ras, and PI3K-Akt pathways; (2) pathways of the comorbidities of BD, including SLE, hepatitis, alcoholism, amebiasis, and melanoma; and (3) common immune, metabolic, and neural-related pathways (Figures 3I and 3J).

To verify the RNA-seq results, we conducted RT-qPCR analysis to evaluate the mRNA level of representative components in the nuclear factor κB (NF-κB) and PI3K-Akt pathways, including Pi3kc2b, Akt1, Traf2, and Traf3 (Figure S6). We observed changes in the expression of these genes that were consistent with the RNA-seq data. Further, in the hippocampus of the Syt7 KO mice, the mammalian target of rapamycin (mTOR) and eukaryotic elongation factor 2 (eEF2) pathways, which contribute to the antidepressant effects of NMDAR antagonists,^25^^,^^26^ both showed opposite changes between the dark and light periods (Figures 3K and 3L). Therefore, insulin likely modulated the same set of downstream factors to adjust the behaviors of the mice. We consider that the signaling pathways shown by BD patients or drug pharmacology (Group 1) might be downstream of the deficits of insulin (and GluN2B-NMDARs) and contribute to the behavioral fluctuations of the Syt7 KO mice. Notably, many of the pathways we detected were autoimmune- and inflammation-related (Group 1–3), indicating that immune responses in the brain probably played important roles in the behavioral abnormalities of the Syt7 KO mice. Moreover, the detection of BD-comorbidity-related pathways (Group 2) might help explain the occurrence of secondary mood-related symptoms in patients suffering from immune diseases such as SLE. The immune disease may activate the signaling molecules downstream of insulin/GluN2B-NMDARs and thus increase the risk of behavioral deficits.

### BDI patient iPSC-derived islet-like organoids show insulin and Syt7 deficits

**Figure 4:**
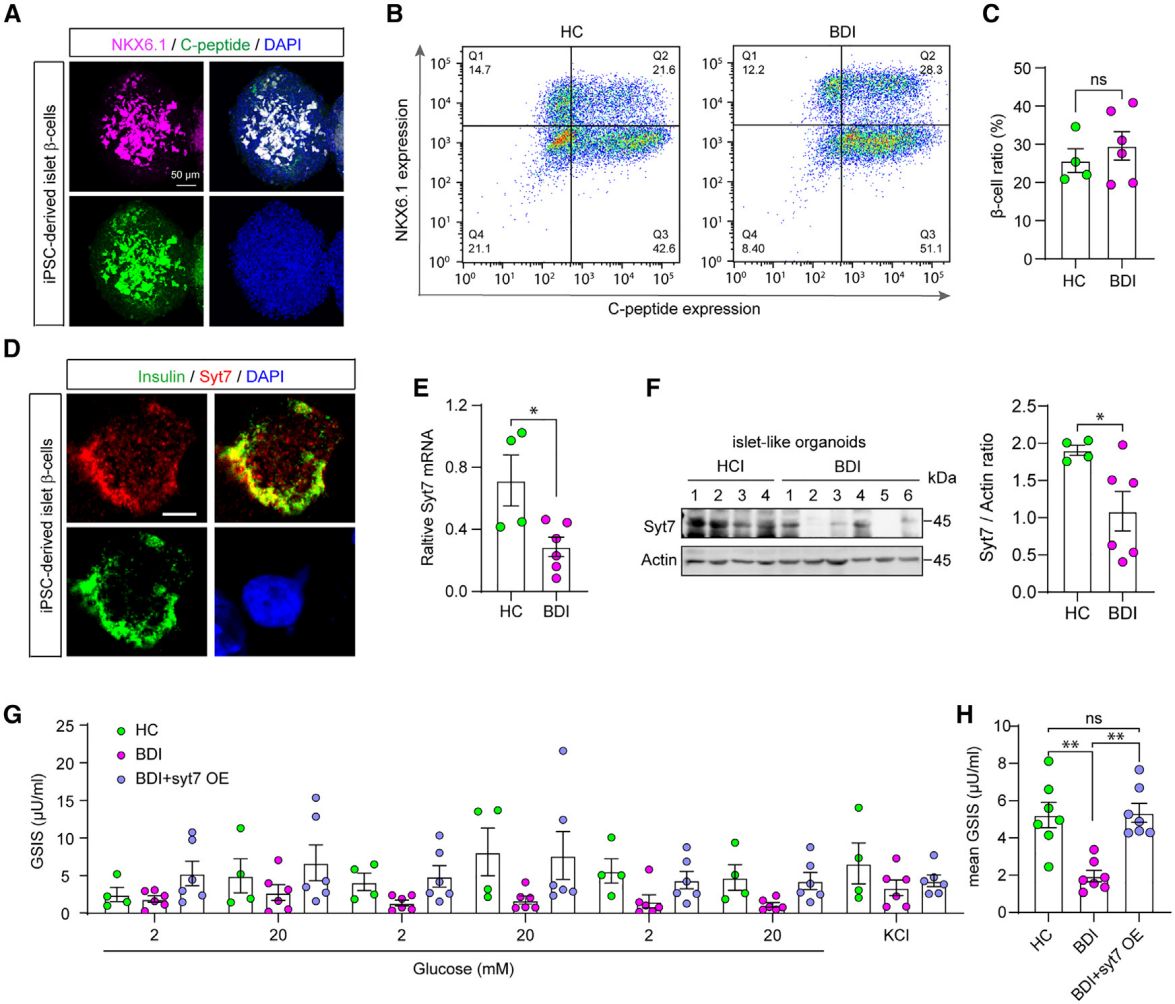
Syt7-dependent insulin hyposecretion in BDI patient iPSC-derived islet β-cells (A) Representative immunostaining images of induced pluripotent stem cell (iPSC)-derived pancreatic islet-like organoids showing a remarkable expression of NKX6.1 (NK6 Homeobox 1) and C-peptide. Scale bar, 50 μm. (B) Sample flow cytometry graphs showing the ratio of NKX6.1^+^/C-peptide^+^ cells of the HC (left) and BDI (right) groups. (C) Quantitative analysis of the ratio of NKX6.1^+^/C-peptide^+^ β-like cells of BDI and HC groups. (D) Representative immunostaining images showing remarkable co-localization of insulin and Syt7 in a differentiated β-like cell. Scale bar, 5 μm. (E) RT-qPCR analysis of Syt7 mRNA expression in BDI iPSC-derived islet-like organoids. (F) Sample immunoblot (left) and quantitative analysis (right) of Syt7 protein expression in BDI iPSC-derived islet-like organoids. (G) Enzyme-linked immunosorbent assay (ELISA) analysis showing the GSIS of BDI iPSC-derived islet-like organoids and organoids expressing exogenous Syt7 through lentiviral infection. Cells were challenged sequentially with 2- and 20-mM glucose for three rounds, with a 30-min incubation for each concentration, followed by a depolarization with 60 mM KCl. *n* = 4 (HC), 6 (BDI), 6 (BDI+Syt7). (H) Mean GSIS level in the iPSC-derived islet-like organoids of BDI patients. (C–F) Student’s t test; (G and H) one-way ANOVA with Sidak’s multiple comparisons test; ∗*p* < 0.05; ∗∗*p* < 0.001; error bars, SEM.

Finally, to investigate whether Syt7 deficits could result in insulin defects in BDI patients, we employed the iPSC model of six BDI patients and four normal healthy controls to generate islet-like organoids that contain insulin-producing β-like cells by employing a recently developed protocol.^1^^,^^21^^,^^27^^,^^28^ Immunofluorescence analysis revealed that the differentiated islet-like organoids showed a decent expression of mature β-cell markers C-peptide and NKX6.1 (Figure 4A). To evaluate the percentage of mature β-cells in the patient and control groups, we performed NKX6.1^+^/C-peptide^+^-based flow cytometry analysis. We found that the two groups showed a similar differentiation ratio of approximately 25% (Figures 4B and 4C). Hence, the iPSCs of BDI patients had a normal ability to differentiate into mature islet β-cells. We investigated the expression and distribution of Syt7 in the islet-like organoids. Immunofluorescence analysis revealed that Syt7 was highly co-localized with insulin in the iPSC-derived islet-like organoids (Figure 4D). Further RT-qPCR and immunoblot analyses revealed that, compared to the healthy controls (HC), the islet-like organoids of the BDI patients showed reduced Syt7 expression (Figures 4E and 4F). This observation is consistent with our previous findings in the iPSC-derived hippocampus-like neurons of these patients and indicates that these patients might have Syt7 deficits in the islet cells.^21^ Moreover, patient iPSC-derived islet-like organoids exhibited a significant decrease in the expression of circadian genes (Figure S4G), which is consistent with our observations in the islets of the Syt7 KO mice. We then performed an ELISA assay to measure the GSIS in the differentiated islet-like organoids. The measured insulin concentration was normalized by the mature β-cell ratio to assay the GSIS level of single cells. Compared to the HC group, the β-like cells of the BDI patients showed reduced GSIS (Figures 4G and 4H), indicating that the patient organoids had defects in insulin secretion. Further, we re-introduced exogenous Syt7 into the islet-like organoids of the BDI patients through lentiviral infection and found that Syt7 expression could increase the GSIS of the patient organoids. Together, our results indicated that the islet-like organoids derived from the BDI patient iPSCs showed Syt7-dependent insulin secretion deficits.

## Discussion

In the present study, we elucidated that Syt7-deficiency-caused insulin deficits induced depression-like behavioral abnormalities in the mice. Furthermore, pancreatic insulin secretion and hippocampal neuronal activity showed opposite diurnal patterns, and thus their disequilibrium in the Syt7 KO mice might contribute to the periodic fluctuation of behaviors.

### Insulin hypoactivity in the hippocampus induces depression-like behavioral deficits

Our results indicated that neuropsychiatric-like behavioral abnormalities could be induced by metabolic abnormalities. It has been reported that more than 10% of BD patients, especially those who have rapid cycling and a more chronic course,^2^^,^^29^^,^^30^ are comorbid with T2D, which is a 3- to 5-fold higher risk compared to the general population.^2^^,^^31^^,^^32^^,^^33^ Even in those BD patients without symptoms of diabetes, more than one-third showed pre-diabetes symptoms.^3^ Several hypotheses have been proposed to explain the comorbidity between these two disorders, such as dysregulated immune-inflammatory responses, insulin-glucose metabolism, and hypothalamic-pituitary-adrenal (HPA) axis functions.^5^ Notably, it has been shown that mice with selective InsR deletion in the brain showed depression-like and anxiety-like behaviors.^34^ Insulin can be released in both the pancreas and the brain.^23^^,^^24^ In the present study, we demonstrated that insulin secretion in the pancreas directly influenced the behaviors of the mice through its signaling in the brain, which, as revealed by our RNA-seq analysis, probably involved downstream autoimmune responses. One interesting observation in our study is that the depression-like behaviors of the Syt7 KO mice could be alleviated by using pharmacological methods to upregulate pancreatic insulin secretion. Previously, clinical research has proposed that insulinopenia might be a feature in the brain of BD patients.^6^^,^^7^ Hence, regulating insulin activity, especially in the brain, might be a potential approach for treating neuropsychiatric-like behavioral abnormalities. In support of this notion, pioglitazone, an anti-diabetic drug, has been clinically used to treat depression and has shown promising efficacies.^3^^,^^8^^,^^9^^,^^10^^,^^11^^,^^12^

In the present study, to investigate the role of insulin signaling in the brain, we used BMS-536924 to block InsRs. However, BMS-536924 can also inhibit insulin-like growth factor receptor (IGF-1R) and thus could not specifically interfere with insulin signaling.^35^^,^^36^ In our experiments, we aimed to test whether insulin can affect depression-like behaviors by blocking the InsRs with BMS-536924. The results showed that BMS-536924 could induce effects opposite to insulin application, thus providing more evidence to support the role of insulin in depression-like behaviors. Nevertheless, we should reiterate that BMS-536924 could have off-target effects, which might influence the observed behavioral outcomes.

In this study, we chose hippocampal DG as the target region for several reasons. First, our previous study has indicated that the DG is involved in mania-like behavioral abnormalities.^21^ Second, previous research has found strong insulin signaling in the granule cell layers (GCLs) of the DG, evidenced by the substantial insulin-1 mRNA signals and *de novo* insulin/C-peptide production in the DG.^24^ Hence, we considered that the DG can reflect the consequences of insulin signaling hypofunction. Third, as the ventral hippocampus relating to depression has been substantially investigated and the molecular and physiological properties of the hippocampus vary considerably along the dorsoventral axis, we were very interested to know whether and how the DG played a role in depressive disorders. Hence, we typically selected the DG to investigate the effects of insulin on mouse behaviors.

### Disequilibrium of hippocampal/pancreatic rhythms induces behavioral fluctuations

Abnormalities in the circadian clock may play a critical role in a number of human disorders. For instance, BD and major depressive disorder (MDD) patients often show a disturbance in sleep.^37^ Consistent with this notion, we have previously observed that more than half of the Syt7 KO animals showed an abnormal circadian rhythm.^21^ As the measurement of GluN2B-NMDAR activity is time-consuming and can hardly be accomplished during a time-sensitive diurnal rhythm test, in the present study, we chose to assay the hippocampal neuronal activity, which can largely determine the activation of GluN2B-NMDARs, to accurately depict the diurnal pattern of GluN2B-NMDARs. Our results indicated that the Syt7-dependent pancreatic and hippocampal activities had opposite diurnal changing patterns, and their balance was likely crucial for stabilizing the behaviors of the mice during a day-night cycle.

One area for improvement in BD research is that the clinical diagnosis of the disease exclusively relies upon the behavioral abnormalities of the patients. It is unclear whether BD is a single disease or a group of disorders that share homologous behavioral features but have differential genetic, molecular, and cellular mechanisms. Previously, we demonstrated that Syt7 had attenuated expression in the plasma samples and iPSC-derived neurons of some patients, and thus Syt7 deficits might contribute to the pathogenesis of a subgroup of BD subjects. However, we should be aware that the Syt7-involved molecular pathway might only represent a subpopulation of BD, and it cannot fully explain the etiology of patients with Syt7 expression defects. The GluN2B/insulin disequilibrium only showed a simplified binary example of how the two opposite behavioral patterns and their fluctuations can be induced by defects in a single gene. Definitely, many factors can contribute to the disease, thus leading to symptomatic diversity in individuals. Moreover, the disease course in the patients is much more complicated than in the mice. For instance, the mood changes in the patients are often experienced within weekly or monthly time frames, and many patients only experience one episode in their lives rather than constantly cycling.

Previously, accumulating evidence has shown that deficiencies in circadian genes can induce mood-related behavioral abnormalities in animal models. For instance, the mutant CLOCK mice exhibited excessive behavioral excitement and mania.^38^^,^^39^^,^^40^ Moreover, BMAL1 knockout cynomolgus monkeys exhibited anxiety and depression, elevated blood cortisol, and defective sensory processing,^41^ and Bmal1 deficiency in mouse cortex can affect depression-like behaviors.^42^ In our study, during the light phase, the Syt7 KO mice exhibited depression-like behaviors, and the expression levels of CLOCK and BMAL1 were elevated, which was consistent with the phenotypes induced by the CLOCK mutant and BMAL1 deficiency. Hence, it is possible that in addition to the insulin hypofunction pathway, circadian gene dysfunction can directly contribute to the behavioral deficits of the Syt7 KO mice. In this study, to accurately monitor the circadian rhythm gene expression pattern, we collected the mouse tissue samples every 3 h and thus set up eight time points within the 24-h period. As this was a time-sensitive experiment, we had to investigate only three mice per group at each time point to maintain the surgery period as short as possible to reduce time-caused variations.

### Immune responses and the HPA axis in BD

Syt7 KO mice have been suggested to exhibit an inflammatory or autoimmune phenotype.^43^ In the present study, we identified pathways related to autoimmune comorbidities of BD, such as SLE and hepatitis, that were changed not only by Syt7 deletion but also by the acute application of Ro25-6981 and BMS-536924 in the brain. Hence, immune responses were likely generated downstream to the insulin/GluN2B signaling in the brain of the Syt7 KO mice, in addition to the peripheral inflammatory abnormalities.^43^ Previously, the neuroinflammation hypothesis has been raised to explain the etiology of BD. In clinical cases, inflammatory disturbances have often been diagnosed in BD patients and used to explain the co-occurrence of BD and major comorbidities, including T2D and cardiovascular diseases. Moreover, clinical studies have also revealed secondary BD occurrence in patients suffering from SLE, hepatitis, alcoholism, amebiasis, and melanoma. Our results might help explain the occurrence of secondary BD symptoms in patients primarily suffering from autoimmune diseases. The immune disease may activate the signaling molecules downstream of insulin/GluN2B-NMDARs and thus increase the risk of mood symptoms in the patients even if they have no functional deficits in insulin or GluN2B-NMDAR activity. This could be one reason why clinical anti-inflammation drugs such as celecoxib, a COX-2 inhibitor, can show efficacy in the clinical treatment of BD.^44^

Dysfunctions in the HPA have been proposed to explain the comorbidity between BD and diabetes.^5^ The glucocorticoid hormone cortisol is a primary product of the HPA axis. Cortisol is one of the most frequently employed biomarkers in psychobiological research, and an elevated cortisol level induced by HPA axis dysregulation has been observed in patients suffering from depression or diabetes.^45^ Previously, it has been suggested that deficits in cortisol level fluctuation from morning to evening could be a mediator between chronic psychosocial stress and poor mental and physical health outcomes.^46^ Hence, the HPA axis and cortisol might be involved in the depression-like behaviors induced by insulin deficits. In the future, it would be interesting to investigate the roles of the HPA axis and cortisol in Syt7-deficit-induced depression-like behaviors and behavioral fluctuation.

### Limitations of the study

Although we have roughly delineated how deficits in insulin signaling contributed to the depression-like behavioral abnormalities and fluctuation phenotype in the Syt7 KO mice, there are several questions and limitations remaining. First, in humans, the circadian rhythms of brain activity and insulin are unlikely to be opposite as they are in mice, even if they are not the same. Hence, the pattern of bipolar behaviors in the patients could not be explained by diurnal changes in the brain/pancreatic activity. The disease itself is affected by many factors, and the disease course in the patients is much more complicated than in the mice. The mood episodes in the patients are often experienced within weekly or monthly time frames, and many patients only experience one episode in their lives rather than constantly cycling. Second, as discussed earlier, the GluN2B/insulin disequilibrium is only a simplified binary example, showing how defects in a single gene can induce two opposite behavioral phenotypes. Syt7 can have other functions with different circadian rhythms. Even in the Syt7 KO mice, the behavioral fluctuating phenotype can be a combined outcome of multiple pathways. The behavioral fluctuating phenotype might only be the 2D projection of the Syt7 function system on the plane of the emotion. The Syt7 function system can project onto the planes of metabolism to contribute to the comorbid metabolic syndrome in BD patients. In the future, it would be interesting to investigate the function of Syt7 in the immune system and its possible contribution to the autoimmune comorbidities of BD. Third, it is unclear which neural circuits can play a crucial role in insulin-hypofunction-induced depression-like behaviors. Fourth, due to the stringent experimental time restrictions, we could only investigate three mice for each group in some experiments. In the future, it would help to investigate more animals to verify our observations further. Fifth, due to the lack of genetic or environmental stress animal models available for BD research, we were unable to employ a BD animal model as a positive control to verify our behavioral observations in the Syt7 KO mice. In the future, further investigations will be necessary to address these questions.

## Resource availability

### Lead contact

Further information and requests for resources and reagents should be directed to and will be fulfilled by the lead contact, Jun Yao (jyao@mail.tsinghua.edu.cn).

### Materials availability

This study did not generate new unique reagents. All key resources are listed in key resources table. Further information and requests for resources and reagents should be directed to the lead contact for non-commercial usage.

### Data and code availability

• RNA-seq data have been deposited at NCBI (GEO Submission [GSE282863], NCBI tracking system #24916721) and are publicly available.
• This paper does not report any original code.
• All other items reported in this paper are available from the lead contact upon request.

## Acknowledgments

We thank all members in the laboratory for helpful discussion and technical assistance. This work was supported by Beijing Municipal Natural Science Foundation (Grant No. Z210011), 10.13039/501100001809National Natural Science Foundation of China (NSFC) (Grant No. 31830038, 32371008), the Open Project of Collaborative Innovation Center for Language Ability of Jiangsu Province, China, and The China Postdoctoral Science Foundation (Grant No. 2022TQ0182 to Y.N.L.).

## Author contributions

Y.N.L. conducted iPSC and imaging experiments. Y.N.L. and W.S. conducted the animal behavioral experiments. Q.W.W. and S.Y.L. conducted patch-clamp recording experiments. C.Y.G., Z.K.X., and S.M. conducted RNA-seq experiments. Y.N.L., C.L., S.S., and S.F.S. conducted molecular biology and biochemistry experiments. Y.N.L., Q.W.W., W.S., and J.Y. analyzed the data. J.Y. and F.H.G. designed the experiments. J.Y. wrote the manuscript with input from all authors.

## Declaration of interests

The authors declare no competing financial interests.

## STAR★Methods

### Key resources table

**Table:** 

REAGENT or RESOURCE	SOURCE	IDENTIFIER
**Antibodies**		

Mouse monoclonal anti-Actin	Abcam	Cat# ab6276; RRID: AB_2223210
Rabbit polyclonal anti-mTOR	Cell Signaling	Cat# 2972; RRID: AB_330978
Rabbit polyclonal anti-*p*-mTOR	Cell Signaling	Cat# 2971; RRID: AB_330970
Rabbit polyclonal anti-eEF2	Cell Signaling	Cat# 2332S; RRID: N/A
Rabbit polyclonal anti-*p*-eEF2	Cell Signaling	Cat# 2331S; RRID: N/A
Rat polyclonal NKX6.1	DSHB	Cat# F55A12-c; RRID: AB_532379
Rabbit polyclonal anti-C-Peptide	Cell Signaling	Cat# 4593S; RRID: AB_10691857
Mouse monoclonal anti-INSRβ	Abcam	Cat# ab69508; RRID: AB_1209215
Rabbit polyclonal anti-*p*-InsR	Abcam	Cat# ab60946; RRID: AB_943587
Mouse monoclonal anti-insulin	Abcam	Cat# ab9569; RRID: AB_296496
Rabbit polyclonal anti-Syt7 antibody	Abcam	Cat# ab106615; RRID: N/A
Goat anti-mouse AleaxFlour488	JacksonImmuno	Cat# 115-546-003; RRID: AB_2338859
Goat anti-rabbit AleaxFlour647	JacksonImmuno	Cat# 111-606-003; RRID: AB_2338079
Goat anti-rabbit ATTO488	Lockland	Cat# 610-152-121S; RRID: AB_10893825
Goat anti-rat Alexa Fluor 568	Abcam	Cat# ab175476; RRID: AB_2813739

**Chemicals, peptides, and recombinant proteins**		

Trypsin-EDTA	Life Technologies	Cat# 25200072
Hank’s Buffered Salt Solution	Life Technologies	Cat# 14175079
BDNF	PeproTech	Cat# 450-02
cAMP	Sigma	Cat# A9501-5G
1 X PBS (Phosphate-buffered saline)	CORNING	Cat# 21-040-CVR
3,3′,5-Triiodo-L-thyronine(T3)	Sigma	Cat# T2877
ALK5 Inhibitor II	Sigma	Cat# R0158-5MG
B-27™ Supplement (50X), Minus VA	Gibco	Cat# 12587010
B-27™ Supplement (50X), serum free	Gibco	Cat# 17504044
BSA	Sigma	Cat# A1470
Chir 99021	Stemgent	Cat# 04-0004
DMEM/F12	Gibco	Cat# C11330500BT
DMEM/F12	Gibco	Cat# 11330032
Dorsomorphin	Sigma	Cat# P5499-5MG
FGF-2	Peprotech	Cat# 100-1813
Gamma-secretase inhibitor XX	Sigma	Cat# SML0649-5MG
GDNF	PeproTech	Cat# AF-450-10
Heparin	Sigma	Cat# H3149
DMSO	Sigma-Aldrich	Cat# D2660
HEPES	Life Technologies	Cat# 15630106
Human Activin A	Peprotech	Cat# 120-14P
Human BMP4	Peprotech	Cat# 120-05
Human FGF10	Peprotech	Cat# 100-26
Human VEGF	Peprotech	Cat# 100-20
Insulin	Sigma	Cat# I9278-5mL
L-AA (L-ascorbic acid)	Sigma	Cat# A0278
Matrigel	Corning	Cat# 354230
Monothioglycerol (MTG)	Sigma	Cat# M6145
*N*-2 Supplement (100X)	Gibco	Cat# 17502048
N-acetyl-L-cysteine	Sigma	Cat# A9165
Neurobasal Medium	Gibco	Cat# 21103049
Nicotinamide	Sigma	Cat# N0636
Opti-MEM Medium	Life Technologies	Cat# 31985088
PEI	Polysciences,Inc.	Cat# 87001-912
Progesterone	sigma	Cat# P0130
PSCeasyII (E8) Medium	Cellapy	Cat# CA1014500
Putrescine	sigma	Cat# P5780
R428 (BGB324)	selleckchem	Cat# ADV947324047
ROCK inhibitor	Axxora	Cat# ALX-270-333
SANT-1	Sigma	Cat# S4572
StemPro Accutase	Gibco	Cat# A11105-01
TGF-b	Peprotech	Cat# 100-21
Trolox	EMD millipore	Cat# 648471
Wnt3a	R&D	Cat# 5036-WN-500
BDNF	PeproTech	Cat# 450-02
RPMI-1640 medium	Gibco	Cat# 11875097
FBS (fetal bovine serum)	Gibco	Cat# 1009141C
Glutamax	Life Technologies	Cat# 35050-061
PEI	Polysciences	Cat# 87001-912
PEG 8000	Beyotime Biotechnology	Cat# ST483
Collagenase IV	ThermoFisher	Cat# 17104-019
BSA	Beijing Biodee Biotechnology Grade	Cat# 0332
Penicillin-Streptomycin	Gibco	Cat# 15140-122
Paraformaldehyde	Sangon	Cat# A500684
NaCl	Sigma-Aldrich	Cat# 746398
D- (+) - Glucose	Sigma-Aldrich	Cat# V900392
KCl	Sigma-Aldrich	Cat# 746436
CaCl2	Sigma-Aldrich	Cat# 793639
MgCl2	Sigma-Aldrich	Cat# 449172
HEPES	Life Technologies	Cat# 15630106
Tris-phosphocreatine	Sigma-Aldrich	Cat# PT1937
EGTA	Tocris	Cat# 2807
Trizol	Life Technologies	Cat# 15596018

**Critical commercial assays**		

Hieff® qPCR SYBR Green Master Mix (No Rox)	YEASEN	Cat# 11201ES08
Mouse Insulin ELISA	Mercodia	Cat# 10-1247-01
SuperScript III Reverse Transcription Kit	ABM	Cat# G492
Pierce™ ECL Western	Thermo Scientific™	Cat# 32106
Cyto-Tune Sendai reprogramming kit	Invitrogen	Cat# A34546

**Experimental models: Cell lines**		

HEK293FT	Invitrogen	R70007; RRID: CVCL_6911

**Experimental models: Organisms/strains**		

Syt7 KO mice	E.R. Chapman (Madison, Wisconsin, USA)	N/A
C57BL/6	Animal Laboratory Center of Tsinghua University	N/A

**Software and algorithms**		

EthoVision XT 11.5	Noldus	https://www.noldus.com/ethovision-xt
NIH ImageJ 1.48	NIH	https://imagej.net/
Graphpad Prism 9.0	GraphPad	https://www.graphpad-prism.cn/
pClamp	Molecular Devices	https://moleculardevices.com.cn/

**Other**		

Microinjection pump	Longer Precision Pump	N/A
Cannula	RWD Life Science	N/A
0.22-μm PVDF filter	Millipore	SLGPR33RB
Bio-Rad CFX96 thermal cycler	Bio-Rad	N/A
FV3000 Confocal	Olymplus	N/A
Olympus FV1200 confocal microscope	Zeiss, Axio Scan.Z1	N/A
VARIOSKAN FLASH multimode reader	Thermo	N/A
RNA-Seq data	NCBI (GEO accession numbers: GSE282863)	

### Experimental model and subject details

#### Statement for identifying the committee approving the experiments

Syt7 KO mice were kindly provided by E.R. Chapman (Madison, Wisconsin, USA) with permission from N.W. Andrews (College Park, MD, USA). All the animal experiments were conducted using 3- to 6-month-old male mice under the guidance and approval of the Institutional Animal Care & Use Committee of Tsinghua University and the Animal Welfare and Ethics Committee of Tsinghua University (Approval ID: 15-YJ2).

The clinical information about the subjects, approving committee, informed consent and clinical trial registration number of the iPSC studies was described in the original articles.^47^^,^^48^ This study included one Li responder in veterans conducted at the University of California, San Diego; the other was the Pharmacogenomics of Bipolar Disorder Study (clinical trial number NCT01272531). All procedures were approved by local human subjects committees. Subjects were initially screened for eligibility, and diagnoses were determined based on the DSM-V criteria. The characteristics of the subjects are detailed in Table S3.

### Method details

#### Plasmids

For the lentiviral experiments, a bicistronic lentiviral vector system, pLox Syn-DsRed-Syn-GFP (pLox), was used by substituting either the dsRed or GFP coding sequence or both with the Syt7 cDNA sequence.

#### Lentivirus preparation and infection

Lentiviral particles were generated by transfecting HEK 293FT cells with virus packaging vectors. HEK 293FT cells were maintained in Dulbecco’s modified eagle medium (DMEM) in 10% FBS, 100 units/ml streptomycin and 100 mg/mL penicillin with 2 mM glutamax (Life Technologies). Transfection was performed using PEI (Polysciences). Five hours after transfection, the medium was changed. Virus supernatant was harvested 60 h post-transfection, filtered with a 0.22 μm PVDF filter (Millipore), ultracentrifuged at 25,000 rpm using a P28S rotor (Hitachi) and stocked in a final volume of 100 μL. The titer of the lentivirus used in all cell culture experiments was at least 5.0 X 10^8^ infectious units (IU) per mL. For *in vivo* stereotactic injection experiments, the lentivirus was further concentrated to a titer of 1.0 X 10^10^ IU/mL.

#### Differentiation of iPSC into islet β-like cells

Differentiation was performed as previously described.^28^ Undifferentiated iPSC lines were regularly tested for pathogens, karyotype and for maintenance of pluripotency markers. For initiation of β-cell differentiation, BDI and HC iPSCs were seeded 1.0 million cells/well in 6-well plates. The six-stage Differentiation was started in 48 h when the iPSC cultures reached 70−80% confluence. Media changes were as follows: for stage 1, Monolayer cultures were treated with RPMI medium (Gibco) containing 100 ng/mL Activin A (Peprotech) and 3μM CHIR990210 (Tocris) for one day (d0-d1). Then they were cultured for 2 days in RPMI medium containing 100 ng/mL Activin A and 5 ng/mL recombinant human bFGF (Peprotech) (d1–d3). During stage 2 (d3-d6), cells were cultured in RPMI with 1% B27 supplement (Life Technologies), 50 ng/mL FGF10 (Peprotech), 0.75 μM dorsomorphin (Sigma), and 3 ng/mL Wnt3a (R&D Systems) for three days. During stage 3 (d7-d8), cells were cultured in medium consisting of DMEM high glucose (Gibco) with 1% B27 supplement, 50 μg/mL ascorbic acid (Sigma), 50 ng/mL Noggin (Peprotech), 50 ng/mL FGF10, and 2 μM all-trans RA (Sigma) for two days. During stage 4 (d8-d13), the medium was changed to DMEM high glucose containing 1% B27 supplement, 50 μg/mL ascorbic acid, 50 ng/mL Noggin, 100 ng/mL EGF (Peprotech) and 10 mM nicotinamide (Sigma), to direct cells toward the PP lineage. During stage 5 (d13-d16), cultures were digested with collagenase in a 37°C incubator for 40–60 min, and then cultured in low attachment plate in MCDB131 medium (Gibco) containing 1 μM T3 (Sigma), 1.5 g/L NaHCO3 (Sigma), 1% L-glutamine (Gibco), 1% B27 supplement (Gibco), 15 mM D-(+)-glucose (Sigma), 10 μg/mL heparin (Sigma), 0.25 μM SANT-1 (Tocris), 100 nM LDN193189 (Cayman), 10 μM ZnSO4 (Sigma), 0.05 μM all-trans RA (Sigma), and 10 μM Y27632 (Tocris). During stage 6, the medium was changed to MCDB131 containing 1 μM T3, 1.5 g/L NaHCO3, 1% L-glutamine, 1% B27 supplement, 15 mM D-(+)-Glucose, 10 μg/mL Heparin, 10 μM Alk5 inhibitor II, 100 nM LDN193189, 10 μM ZnSO4, and 100 nM gamma-secretase inhibitor XX (Tocris) for seven days (d16-d23). Then the medium was changed to 100 nM LDN193189 and 100 nM gamma-secretase inhibitor XX with trolox and R428 for 15days (d23-d38). Cell clusters were collected at day 38 for ELISA and qRT-PCR analysis or dissociated with trypsin for flow cytometry or fixed in 1.6% PFA for immunohistochemistry analysis.

#### Differentiation of iPSC into DG-like neurons

The forebrain neural progenitor cells (NPCs) derived from BDI patients and healthy people were characterized as previously described.^27^^,^^48^ The information about the approving committee, informed consent and clinical trial registration number of the iPSC studies was described in the original articles.^27^^,^^48^ To obtain hippocampal DG-like neurons, NPCs were differentiated in DMEM/F12 supplemented with N2 (Life Technologies), B27 (Life Technologies), 20 ng/mL BDNF (Peprotech), 1 mM dibutyryl-cyclicAMP (Sigma), 200 nM ascorbic acid (Sigma), 1 μg/mL Laminin, and 620 ng/mL Wnt3a (R&D) for three to four weeks. Wnt3a was removed after three weeks. All cells used in the present study were verified as mycoplasma contamination free.

#### Mouse pancreatic islet isolation and β-cell culture

Islets were isolated in the light phase by using 0.5 mg/mL of collagenase (Sigma) before digestion at 37°C for 10 min. Then islets were cultured overnight at 11 mM glucose in RPMI-1640 medium supplemented with 10% (vol/vol) FBS and 1% (vol/vol) penicillin/streptomycin. Islets were dissociated into single β-cells using trypsin digestion for 5 min at 37°C and allowed to attach to poly-D-lysine-coated 24-well culture plates.

#### Mouse primary neuronal culture

Mouse hippocampal neurons were dissected from newborn WT and homozygous Syt7 KO mice in the dark phase and incubated in 0.25% trypsin-EDTA (Life Technologies) for 15 min at 37°C. After washing with Hank’s Buffered Salt Solution plus 5 mM HEPES (Life Technologies), 20 mM D-glucose and 2% fetal bovine serum (FBS) (Gibco), the neurons were mechanically dissociated in culture medium and plated on poly-D-lysine-coated glass coverslips at a density of 50,000–100,000 cells/cm^2^. Cells were grown in Neurobasal-A medium (Life Technologies) supplemented with 2% B-27 (Life Technologies) and 2 mM glutamax (Life Technologies). Cultures were maintained at 37°C in a 5% CO_2_-humidified incubator.

#### Insulin test

After the mice were fasted overnight with free access to water, blood samples were collected to determine the peripheral insulin level. After injection of glucose (2 mg/g body weight) or equal volume PBS, blood samples were collected from the tail vein at each time point for each tested mouse to determine the peripheral insulin level. Plasma for insulin measurement was obtained from ∼50 μL of collected blood. Blood samples were mixed with 2 μL of 0.5 M EDTA on ice and centrifuged at 1,000 g for 10 min. Plasma insulin concentrations were determined using the Ultrasensitive Mouse Insulin ELISA kit (Mercodia).

A GSIS test in cultured cells was performed as described previously.^49^ Briefly, cells were rinsed twice and preincubated in low (2 mM) glucose Krebs buffer for 2 h to remove residual insulin. Cells were then washed two times in Krebs buffer and incubated in 5 mM glucose Krebs buffer for 30 min, and supernatant collected. Then cells were washed two times in Krebs buffer and incubated in 20 mM glucose Krebs buffer for 30 min, and supernatant was collected. This sequence was repeated two additional times. Finally, cells were incubated in Krebs buffer containing 2 mM glucose and 30 mM KCl for 30 min and then supernatant collected. Supernatant samples containing secreted insulin were processed using the Ultrasensitive Mouse Insulin ELISA kit (Mercodia).

#### Housing conditions of the animals

The mice were housed under a 12-h light/dark cycle, with light from 19:00 (ZT0) to 07:00 (ZT12), maintaining an ambient illumination of approximately 200 lux. The environmental conditions were controlled to a temperature of 22 ± 2°C and a relative humidity of 45%–65%, with continuous access to food and water. To promote normal social interactions, we housed 5–6 mice per cage under standard feeding conditions. Prior to the forced swim test (FST), learned helplessness test (LH), and light/dark box (LDB) test, the animals were acclimatized to the testing room for at least 1 h to adapt to the experimental environment. For the Sucrose Preference Test, it was necessary to house each mouse individually to monitor water intake accurately. After completing the tests, the mice were promptly returned to their standard housing conditions.

#### Behavioral assays

Syt7 KO mice were kindly provided by E.R. Chapman (Madison, Wisconsin, USA) with permission from N.W. Andrews (College Park, MD, USA). For all behavioral tests, the experimenter was blinded to the genotype of mice, and all mice were randomly tested. The sample size was determined through random allocation based on the underlying test. Animals that were sick or failed to be infected with lentivirus were excluded from the data analysis. Otherwise, no data were excluded. All the animal experiments were conducted using 3- to 6-month-old male mice under the guidance and approval of the Institutional Animal Care & Use Committee of Tsinghua University and the Animal Welfare and Ethics Committee of Tsinghua University (Approval ID: 15-YJ2). WT siblings with the similar age were used as a control for the Syt7 KO mice. Different groups of mice were used for dark phase and light phase testing. Activity was recorded by a suspended digital camera and analyzed by EthoVision XT 11.5 (Noldus) after 30 min habituation in the test room (600 lx) in light/dark box test and open field test. The same parameter setting for the definition of each behavior was applied for all the mice tested in different behavior tests. Following behavioral tests, animals infused with virus were dissected to perform immunostaining analysis to verify virus infection, and animals without appropriate virus infection were excluded from analysis. Animals that accidentally died during the study were included in analysis for all completed tests. The data were analyzed by EthoVision XT 11.5 software (Noldus).

Forced swim test (FST): Animals were transferred to the test room at least 1 h before the beginning of the test to adapt to the experimental environment in advance. Animals were individually introduced to a cylinder (20 cm diameter × 30 cm height) filled with 15 cm of water (23 ± 1°C) and swam for 6 min under normal light. Then, immediately remove the mice from the cylinder, dry the mice with absorbent paper, and put an appropriate amount of absorbent paper in the feeding cage of the mice to help keep them warm. Immobility time was defined as the time when animals remained floating or motionless that necessary to keep balance in the water. Data acquisition and analysis were carried out by trained individuals blind to the genotype and treatment during the test.

Sucrose preference test (SPT): Mice were individually held on a 12:12 light/dark cycle with food and water and with the temperature at 22 ± 2°C. The protocol of the sucrose preference test consisted of three procedures: adaptation, water deprivation, and the preference test. During adaptation, mice were placed individually in their home cages are given 48 h of continuous exposure to two regular bottles filled with regular water, followed by two regular bottles filled with 2% sucrose solution for another 48 h of continuous exposure. During the water deprivation, mice were deprived of water for 24 h. Immediately after deprivation, all animals were given 12-h access to one tube of 2% (wt/vol) sucrose solution and one tube of regular water. Each tube was weighed before and after the test. The position of the sucrose and water bottles was exchanged every 6 h. At the end of the test, all animals were returned to group housing with adequate food and water. Sucrose preferences were calculated as follows: sucrose consumed/(sucrose consumed + water consumed).

Learned helplessness test (LH): The learned helplessness model consisted of three different phases: inescapable shock training, learned helplessness screening, and the test. Mice were placed in one chamber of two-chamber shuttle boxes (MedAssociates) for a 5-min adaptation period before the test. During the inescapable shock training phase (day 1–3), the mice received 120 inescapable foot-shocks (0.45 mA, 15 s duration, randomized average inter-shock interval 45 s) with the door closed between the two chambers of the shuttle boxes each day. For the learned helplessness screening phase (day 4), the door was raised at the onset of the shock (0.45 mA) and the shock ended either when the mouse stepped through to the other side of the shuttle boxes or after 3 s. Mice that had more than 5 escape failures developed helplessness behavior during the 10 screening shocks. On the test day, the animals were placed in the shuttle boxes and 0.45-mA shocks were delivered concomitantly with door opening for the first 5 trials, followed by a 2-s delay for the next 40 trials and inter-trial intervals were randomized at an average of 30 s. The shock was terminated either when the animal crossed over to the second chamber or after 24 s. Latency to step through the door and the number of escape failures were recorded for the last 40 trials by automated computer software (MED-PC IV).

Light/dark box (LDB) test: Mice were placed singly in the dark side of the light/dark box apparatus and allowed to move freely for 10 min; the time spent in the light box was analyzed.

#### Osmotic minipump infusion, cannulation surgery and intraperitoneal injection

Surgery was performed as described previously.^50^ Briefly, animals were anesthetized with a mixture of oxygen and isoflurane and surgically implanted with two guide cannulas (RWD Life Science, China) targeting the bilateral hippocampal DG according to bregma (DG: coordinates AP +2.0, ML ±1.5 mm and DV -2.5 mm relative to the bregma). Mice were allowed to recover from surgery for at least 7 days before behavioral tests or molecular assessments. At specific time points during the behavioral or molecular tests, the mice were bilaterally infused with 1.5 μL insulin (10 UI/ml), BMS-536924 (750 mM; Sigma Aldrich), or vehicle (0.4% dimethyl sulfoxide) using two microinjection pumps (Harvard Apparatus) at a flow rate of 0.1 μL/min into each hemisphere, simultaneously. For the intraperitoneal injection, insulin, repaglinide and octreotide were dissolved in saline to a final concentration of 2 UI/kg, 1.25 mg/kg, and 0.125 mg/mL, respectively. The drug solutions or vesicle control were injected intraperitoneally 30 min before the behavioral tests at a dose of 10 mL/kg.

#### Whole-cell patch clamp recording

Whole-cell recordings were performed in voltage-clamp mode using a MultiClamp 700B amplifier (Molecular Devices). For acute slice preparation, mice were euthanized under Pentobarbital Sodium. Brains were removed and placed in ice-cold solution containing (in mM): 110 choline chloride, 25 NaHCO_3_, 7 MgCl_2_, 2.5 KCl, 1.3 NaH_2_PO_4_, 0.5 CaCl_2_, 1.3 Na-ascorbate, 0.6 Na-pyruvate and 20 D-glucose. 300-μm-thick slices for hippocampus were prepared on a Compresstome VF-330 vibratome (Precisionary Instruments). Slices were transferred for 60 min to 33°C artificial cerebrospinal solution (ACSF) containing (in mM): 124 NaCl, 26 NaHCO_3_, 10 glucose, 3 KCl, 2 CaCl_2_, 1.25 KH_2_PO_4_ and 1 MgCl_2_. Whole-cell patch-clamp recordings were performed from hippocampus slice at 33 ± 1°C with flow rates of 2 mL/min in ACSF containing 2mM CaCl_2_. Patch pipettes were pulled from borosilicate glass and had resistances of 3–5 MΩ when filled with internal pipette solution (130 mM K-gluconate, 1 mM EGTA, 5 mM Na-phosphocreatine, 2 mM Mg-ATP, 0.3 mM Na-GTP, 10mM HEPES; pH 7.3). The membrane potential was held at −70 mV. For current-clamp recordings, a hyperpolarized current was injected into the neuron to a membrane potential of −55 mV for evoked APs or −45 mV for spontaneous APs. Data were acquired using pClamp10 software (Molecular Devices), sampled at 10 kHz, and filtered at 2 kHz. Offline data analysis was performed using Clampfit software (Molecular Devices).

#### Immunoblot analysis

Neurons were lysed in RIPA buffer (50 mM Tris-Cl, pH 8.0, 150 mM NaCl, 1% Nonidet P-40, 0.5% sodium deoxycholate and 0.1% SDS) plus a complete protease inhibitor cocktail (Roche). Lysates were centrifuged and supernatants were subjected to SDS-PAGE. The blots were developed using an ECL kit (Pierce). Protein levels were quantified by densitometry using NIH ImageJ 1.48 software. Primary antibodies were as follows: mouse monoclonal anti-Actin antibody (1:5000, Abcam, #ab6276), rabbit polyclonal anti-mTOR antibody (1:500, Cell Signaling, #2972), rabbit polyclonal anti-*p*-mTOR antibody (1:500, Cell Signaling, #2971), rabbit polyclonal anti-eEF2 antibody (1:1000, Cell Signaling, #2332S), rabbit polyclonal anti-*p*-eEF2 antibody (1:1000, Cell Signaling, #2331S).

#### RNA-seq analysis

RNA was prepared into RNA-Seq libraries using an Illumina TruSeq Stranded Total RNA Sample Prep Kit with Ribo-zero Gold (Human/Mouse/Rat) (Illumina) according to the standard procedures. Total RNA-Seq libraries were sequenced paired-end 2 × 100 base pairs (bp) using the Illumina HiSeq 2500 platform according to the manufacturer’s specifications. Low-quality reads and readthrough adaptor sequences were trimmed using SOAPnuke, version 1.5.2. The trimmed reads were mapped to the mouse genome (mm10/GRCm38) using HISAT2, version 2.0.4. The assignment of reads to gene regions was performed using Bowtie2, version 2.2.5. Quantitative analysis of gene expression was performed using RSEM, version 1.2.12. DAVID (http://david.abcc.ncifcrf.gov/) was used to perform the gene functional annotation analysis. The Kyoto Encyclopedia of Genes and Genomes (KEGG) and Ingenuity Pathway Analysis (IPA) were chosen as the background database for enriched pathway analysis. BD-related gene IDs were searched using GeneCards (http://www.genecards.org/).

#### Immunofluorescence

Mice were euthanized two weeks after viral delivery and transcardially perfused with 4% PFA in PBS. Fixed tissue was sectioned with 50-μm thickness using vibratome (Leica, VT1000S). Then antigens were retrieved by incubating for 10 min in 100 mM Tris (pH 7.4). Sections were blocked with 5% normal goat serum (NGS) in TBST (137 mM NaCl, 20 mM Tris pH 7.6, 0.05% Tween 20) for 1 h and incubated with primary antibodies overnight at 4°C. After three washes in TBST, samples were incubated with secondary antibodies. Following three washes with TBS, cells were incubated with DAPI (0.1 μg/mL, Sigma) for 15 min, followed by three washes with TBS to remove DAPI. Fluorescent signals were detected on an Olympus FV1200 confocal microscope by sequential acquisition or on slide scanner (Zeiss, Axio Scan.Z1) and images were processed using ImageJ 1.48 software (NIH). Primary antibodies were as follows: rat polyclonal NKX6.1 antibody (1:200, DSHB, #F55A12-c), rabbit polyclonal anti-C-Peptide antibody (1:200, Cell Signaling Technology, #4593S), mouse monoclonal anti-INSRβ (1:500, Abcam, #ab69508), rabbit polyclonal anti-*p*-InsR antibody (1:500, Abcam, #ab60946), mouse monoclonal anti-insulin antibody (1:200, Abcam, #ab9569) and rabbit polyclonal anti-Syt7 antibody (1:200, Abcam, #ab106615). Secondary antibodies were as follows: goat anti-mouse AleaxFlour488 antibody (1:500, JacksonImmuno, #115-546-003), goat anti-rabbit AleaxFlour647 antibody (1:500, JacksonImmuno, #111-606-003), goat anti-rabbit ATTO488 antibody (1:500, Lockland, #610-152-121S) and goat anti-rat Alexa Fluor 568 antibody (1:500, Abcam, #ab175476).

#### Quantitative reverse transcription PCR

Total RNA was isolated using Trizol (Life Technologies) according to the manufacturer’s instructions. cDNA was synthesized using SuperScript III Reverse Transcription Kit (Life Technologies), and quantitative reverse transcription PCR (qRT-PCR) was performed on a Bio-Rad CFX96 thermal cycler using SYBR green supermix (Bio-Rad) and gene-specific primers. The primer sequences are listed in Table S4. Quantitative analysis was performed employing the ΔΔCT method and the GAPDH as the endogenous control.

### Quantification and statistical analysis

Data are shown as mean values ±SEM. For two-group comparisons, the F-test was used to assess the equality of variances, and the two-tailed unpaired Student’s t test was used, depending on the equality of variances. One-way ANOVA test with Tukey’s multiple comparisons test and two-way ANOVA with Sidak’s multiple comparisons test were used for multiple comparisons. Statistical details of experiments and analyses can be found in the figure legends and main text above. A *p* value >0.05 was considered not significant (n.s.). Statistical significance was evaluated at *p* < 0.05, ∗*p* < 0.05; ∗∗*p* < 0.001. GraphPad Prism 9 was utilized for all statistical analyses (GraphPad Software). The analysis approaches have been justified as appropriate by previous biological studies.
