# Supplementary material for: Synaptotagmin-7 deficit causes insulin hypoactivity and contributes to behavioral alterations in mice
Source: iScience. 2025 Apr 4;28(5):112354. doi: 10.1016/j.isci.2025.112354 (PMC12053657; doi:10.1016/j.isci.2025.112354)
Supplement: Document S1. Figures S1–S6 and Tables S1–S4 [file mmc1.pdf]

iScience, Volume *28*

Supplemental information

Synaptotagmin-7 deficit causes insulin hypoactivity and contributes to behavioral alterations in mice

Yao-Nan Liu, Qiu-Wen Wang, Si-Yao Lu, Wei Shen, Chongye Guo, Zhikai Xing, Chang Li, Shan Sun, Sen-Fang Sui, Shuangli Mi, Fred H. Gage, and Jun Yao

Supplementary Information for

Synaptotagmin-7 deficit causes insulin hypoactivity and contributes to behavioral alterations in mice

Yao-Nan Liu[1], Qiu-Wen Wang[2], Si-Yao Lu[3], Wei Shen[1], Chongye Guo[4], Zhikai Xing[4], Chang Li[5], Shan Sun[5], Sen-Fang Sui[5], Shuangli Mi[4], Fred H. Gage[2], and Jun Yao[1]

[1] State Key Laboratory of Membrane Biology, Tsinghua-Peking Center for Life Sciences, School of Life Sciences, Tsinghua University, Beijing 100084, China.
[2] Laboratory of Genetics, The Salk Institute for Biological Studies, La Jolla, CA 92037, USA.
[3] Jiangsu Key Laboratory of Language and Cognitive Neuroscience, School of Linguistic Sciences and Arts, Jiangsu Normal University, Xuzhou 221116, China; Jiangsu Collaborative Innovation Center for Language Ability, Xuzhou 221009, China.
[4] Key Laboratory of Genomic and Precision Medicine, Beijing Institute of Genomics, Chinese Academy of Sciences / China National Center for Bioinformation, Beijing 100101, China; University of Chinese Academy of Sciences, Beijing 100049, China.
[5] State Key Laboratory of Membrane Biology; Beijing Frontier Research Center for Biological Structure; School of Life Sciences, Tsinghua University; Beijing 100084, China.

Please address correspondence to: Jun Yao (jyao@mail.tsinghua.edu.cn).

This PDF file includes:

SUPPLEMENTAL MATERIALS

Figure S1 to S6.
Table S1 to S4.

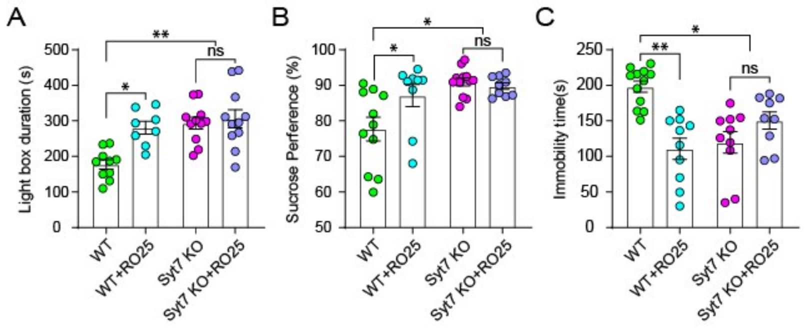

Fig. S1. GluN2B-NMDAR antagonist Ro25-6981 has moderate effects on the mania-like behavioral abnormalities of Syt7 KO mice in the dark phase. (**A**) Effects of Ro25-6981 (RO25) on the light/dark box (LDB) time-in-light of wild type (WT) and Syt7 KO mice in the dark phase. n = 10/8/11/11. (**B**) Effects of RO25 on sucrose preference ratio. n = 11/9/12/9. (**C**) Forced swim test (FST) immobility time of mice treated with Ro25-6981. n = 12/10/10/9. Compared to the WT mice, the Syt7 KO mice showed increased sucrose preference ratio and LDB time-in-light and decreased FST immobility time. Moreover, RO25 showed obvious antidepressant effects on the WT mice but not on the Syt7 KO mice. One-way ANOVA test with Tukey's multiple comparisons test. *$P < 0.05$; **$P < 0.001$; error bars, s.e.m.

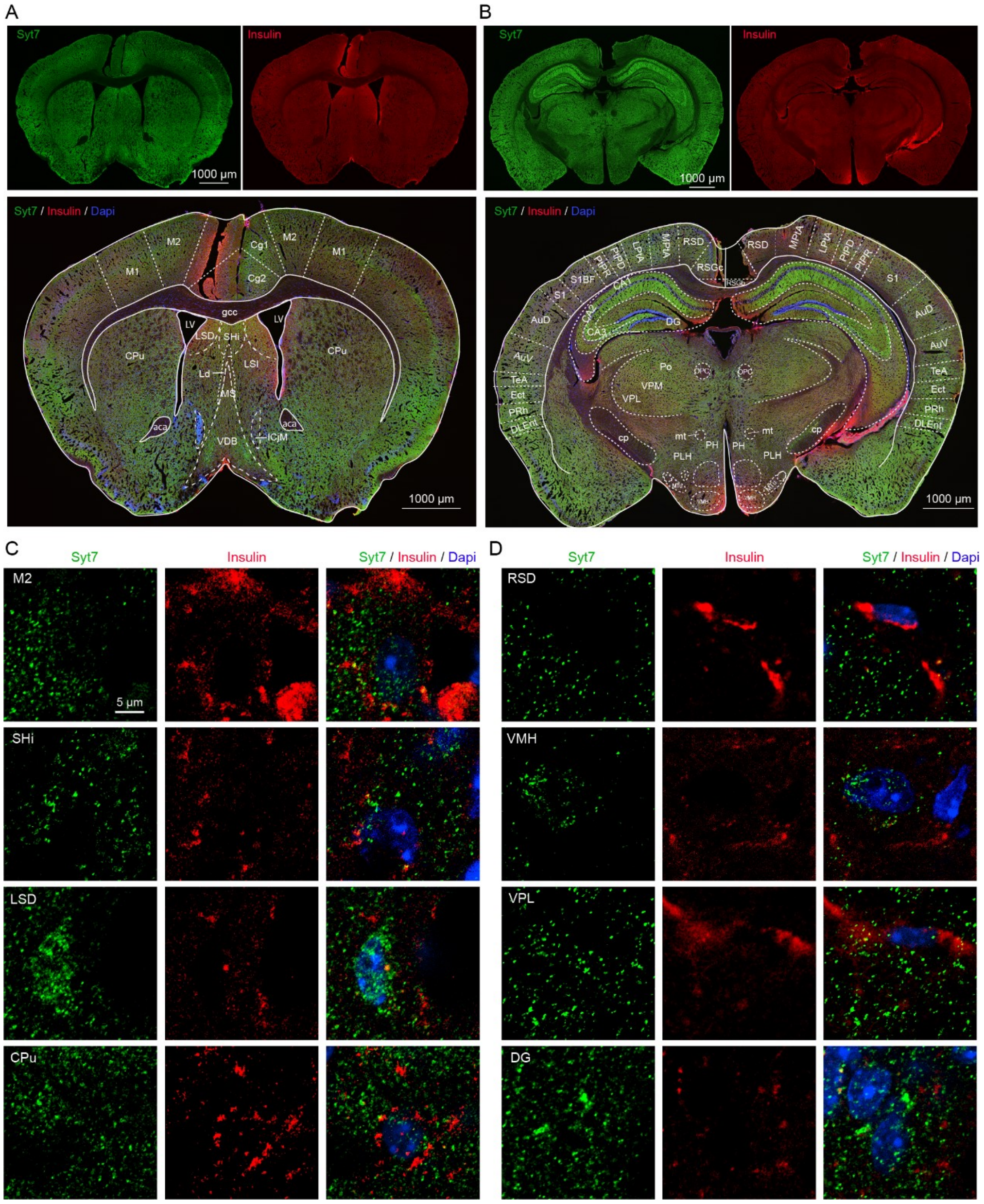

Fig. S2. Insulin activity in the mouse brain. (**A-B**) Representative immunostaining images showing double-staining of insulin and Syt7 in mouse brain slices (low magnification). Scale bar, 1000 μm. (**C**) Representative immunostaining images showing the double-staining of insulin and Syt7 in the M2, SHi, LSD, and CPu. The Images were from the panel A (high magnification). Scale bar, 5 μm. (**D**) Representative immunostaining images showing the double-staining of insulin and Syt7 in the RAD, VMH, VPL, and DG. Images were from the panel B (high magnification). Scale bar, 50 μm. Note: M2, secondary motor cortex; Shi, septohippocampal nucleus; LSD, lateral septal nucleus, dorsal part; CPu, caudate putamen (dorsal striatum); RSD, retrosplenial dysgranular cortex; VMH, ventromedial hypothalamic nucleus; VPL, ventral posterolateral thalamic nucleus; DG, dentate gyrus.

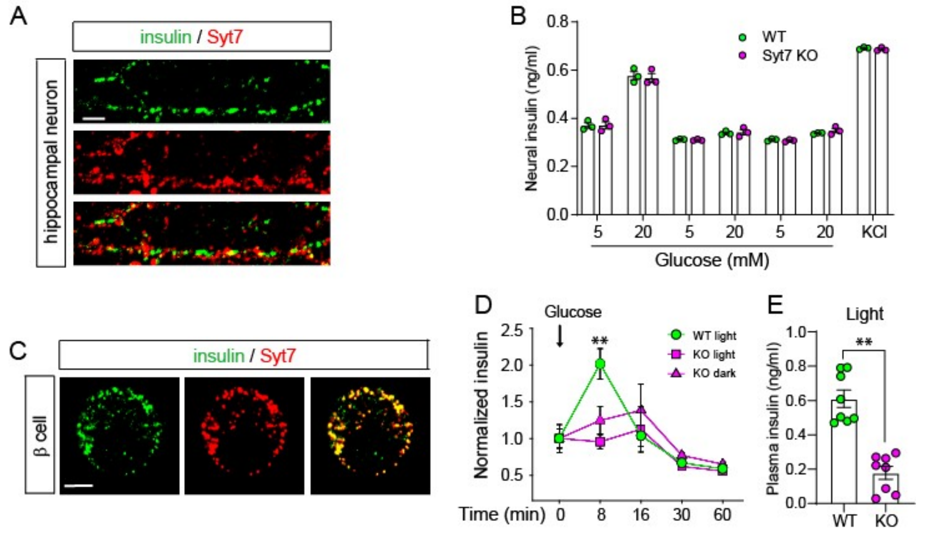

Fig. S3. Insulin activity in the mouse brain and islets. (**A**) Representative immunostaining images showing sparse co-localization of insulin and Syt7 in cultured hippocampal neurons. Scale bar, 5 µm. (**B**) Enzyme-Linked Immunosorbent Assay (ELISA) analysis showing similar levels of glucose-stimulated insulin secretion (GSIS) in the WT and Syt7 KO hippocampal neurons. Neurons were challenged sequentially with 5 and 20 mM glucose for three rounds, with a 30-minute incubation for each concentration, followed by a depolarization with 60 mM KCl. (**C**) Immunostaining images showing remarkable co-localization of insulin and Syt7 in isolated pancreatic β-cells. Scale bar, 5 µm. (**D**) GSIS in WT and Syt7 KO mice in the dark or light phase. n = 8. (**E**) Overall insulin secretion in the light phase. n = 8. Student's *t*-test; *$P < 0.05$; **$P < 0.001$; error bars, s.e.m.

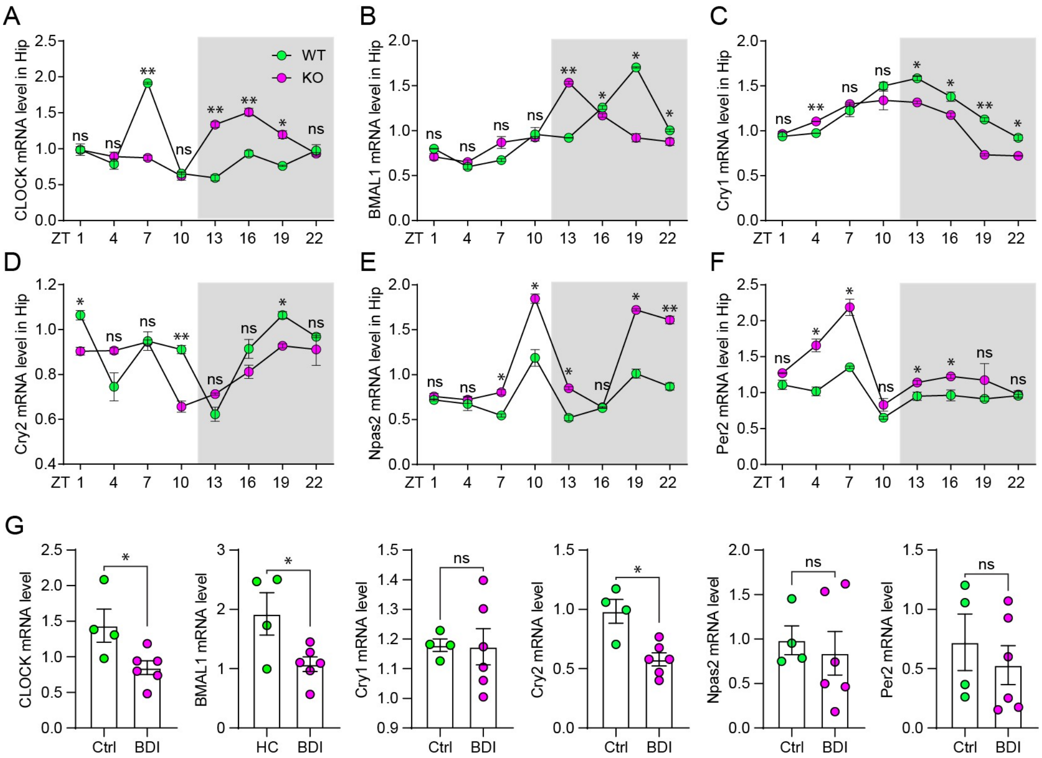

Fig. S4. Distinct circadian rhythms of hippocampal neurons and BDI iPSC-derived islet-like organoids. (**A-F**) Circadian expressing patterns of circadian genes in the hippocampus of Syt7 KO mice. The circadian genes include CLOCK (**A**), BMAL1 (**B**), Cry1 (**C**), Cry2 (**D**), Npas2 (**E**), and Per2 (**F**)). n = 3. (**G**) mRNA expression of circadian genes (including CLOCK, BMAL1, Cry1, Cry2, Npas2, and Per2) in the islet-like organoids of healthy control (HC) and bipolar I disorder (BDI) patients. n = 4 (HC), 6 (BDI). Note: CLOCK, Clock Circadian Regulator; BMAL1, Basic Helix-Loop-Helix ARNT Like 1; Cry1, Cryptochrome Circadian Regulator 1; Cry2, Cryptochrome Circadian Regulator 2; Npas2, Neuronal PAS Domain Protein 2; Per2, Period Circadian Regulator 2. (**A-F**) Two-way ANOVA with Sidak's multiple comparisons test; (**G**) Student's *t*-test; *$P < 0.05$; **$P < 0.001$; error bars, s.e.m.

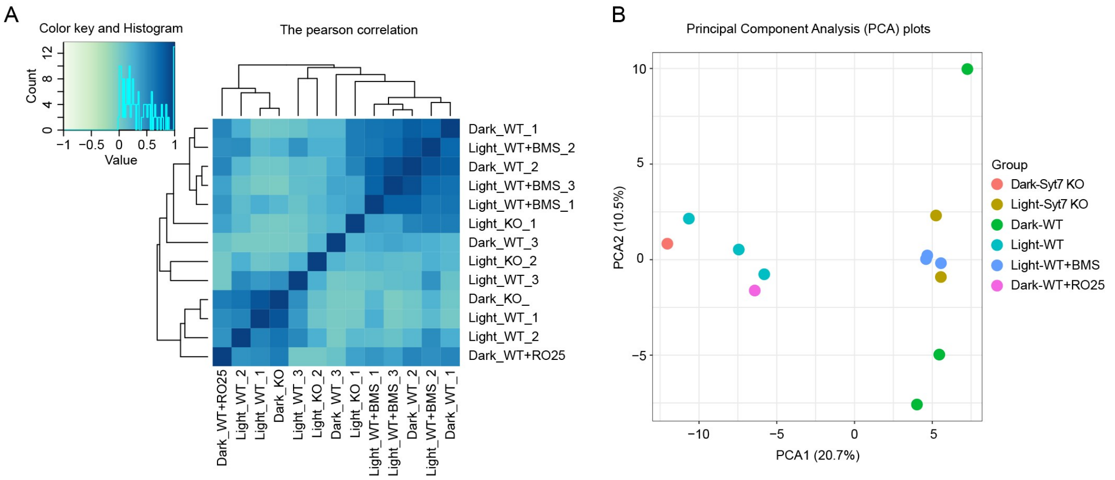

Fig. S5. Sample distance plots and PCA plots for all datasets. (**A**) Heatmap showing the Spearman's correlation coefficient of mouse groups, including WT, Syt7 KO, WT+Ro25, and WT+BMS. Each column/row represents one sample. Color scale: 0.0 (lighter green color) to 1.0 (darker blue color). (**B**) Principal Component Analysis (PCA) plots (**C**) for all datasets, including WT+Ro25, KO, WT and WT+BMS.

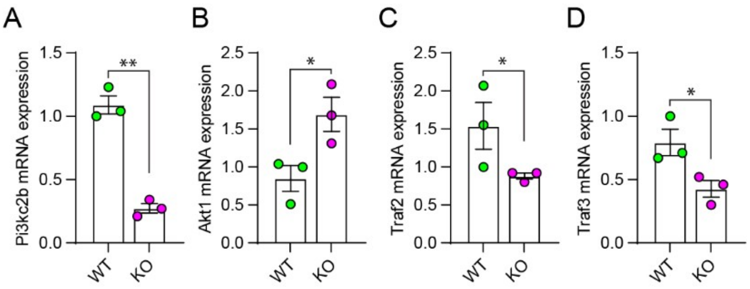

Fig. S6. mRNA levels of the downstream in the hippocampus of Syt7 KO mice. (**A-D**) mRNA levels of Phosphatidylinositol-4-Phosphate 3-Kinase Catalytic Subunit Type 2 Beta (Pi3kc2b) (**A**), AKT Serine/Threonine Kinase 1 (Akt1) (**B**), TNF Receptor Associated Factor 2 (Traf2) (**C**) and TNF Receptor Associated Factor 3 (Traf3) (**D**) in the hippocampus of WT and Syt7 KO mice. n = 3 mice. (**A-D**) Student's t-test; *P < 0.05; **P < 0.001; error bars, s.e.m.

Table S1. KEGG pathways showing bi-directional differential expression in the hippocampus of manic and depressive Syt7 KO mice.

KEGG ID	Term	Count	%	p value
mmu00230	Purine metabolism	41	1.3800	4.62E-06
mmu04380	Osteoclast differentiation	30	1.3239	1.26E-05
mmu05140	Leishmaniasis	19	0.8385	3.44E-05
mmu05133	Pertussis	19	0.8385	0.000266
mmu05144	Malaria	14	0.6178	0.000624
mmu04390	Hippo signaling pathway	31	1.0434	0.000626
mmu05168	Herpes simplex infection	37	1.6328	0.000675
mmu05203	Viral carcinogenesis	40	1.7652	0.000679
mmu05150	Staphylococcus aureus infection	14	0.6178	0.00095
mmu05322	Systemic lupus erythematosus	28	1.2357	0.001247
mmu05169	Epstein-Barr virus infection	37	1.6328	0.001256
mmu04514	Cell adhesion molecules (CAMs)	30	1.3239	0.001275
mmu05217	Basal cell carcinoma	15	0.5049	0.001628
mmu00220	Arginine biosynthesis	8	0.2693	0.002587
mmu00340	Histidine metabolism	9	0.3029	0.002675
mmu04610	Complement and coagulation cascades	17	0.7502	0.002935
mmu05134	Legionellosis	14	0.6178	0.003406
mmu04010	MAPK signaling pathway	40	1.7652	0.003774
mmu05161	Hepatitis B	26	1.1474	0.004911
mmu05205	Proteoglycans in cancer	33	1.4563	0.005914
mmu05142	Chagas disease	20	0.8826	0.00595
mmu04672	Intestinal immune network for IgA production	11	0.4854	0.007085
mmu05202	Transcriptional misregulation in cancer	28	1.2357	0.007229
mmu05412	Arrhythmogenic right ventricular cardiomyopathy (ARVC)	16	0.5385	0.007670
mmu04621	NOD-like receptor signaling pathway	13	0.5737	0.008133
mmu04020	Calcium signaling pathway	31	1.0434	0.009801
mmu04620	Toll-like receptor signaling pathway	19	0.8385	0.010451
mmu04668	TNF signaling pathway	20	0.8826	0.010955
mmu05166	HTLV-I infection	41	1.8094	0.010958
mmu04550	Signaling pathways regulating pluripotency of stem cells	25	0.8415	0.011898
mmu04932	Non-alcoholic fatty liver disease (NAFLD)	26	1.1474	0.012353
mmu00240	Pyrimidine metabolism	19	0.8385	0.012732
mmu01130	Biosynthesis of antibiotics	35	1.1781	0.012799
mmu00531	Glycosaminoglycan degradation	7	0.3089	0.014229
mmu04612	Antigen processing and presentation	16	0.7061	0.014702
mmu04310	Wnt signaling pathway	25	0.8415	0.015327
mmu04916	Melanogenesis	19	0.6396	0.017777
mmu04350	TGF-beta signaling pathway	17	0.5722	0.018068
mmu00410	beta-Alanine metabolism	9	0.303	0.021132
mmu04145	Phagosome	27	1.1915	0.023324
mmu05416	Viral myocarditis	15	0.662	0.023371

mmu04068	FoxO signaling pathway	22	0.9709	0.024338
mmu04977	Vitamin digestion and absorption	7	0.2356	0.025317
mmu03020	RNA polymerase	8	0.3530	0.025411
mmu00240	Pyrimidine metabolism	19	0.6395	0.02582
mmu04022	cGMP-PKG signaling pathway	28	0.9424	0.026506
mmu05152	Tuberculosis	27	1.1915	0.026554
mmu01100	Metabolic pathways	148	6.5313	0.028755
mmu03030	DNA replication	9	0.3029	0.029578
mmu03410	Base excision repair	9	0.3029	0.029578
mmu05321	Inflammatory bowel disease (IBD)	12	0.5295	0.029827
mmu04064	NF-kappa B signaling pathway	17	0.7502	0.029965
mmu00280	Valine, leucine and isoleucine degradation	12	0.4039	0.030433
mmu05332	Graft-versus-host disease	11	0.4854	0.030791
mmu04976	Bile secretion	14	0.4712	0.038170
mmu04922	Glucagon signaling pathway	17	0.7502	0.038476
mmu04913	Ovarian steroidogenesis	12	0.4039	0.038562
mmu04923	Regulation of lipolysis in adipocytes	12	0.4039	0.038562
mmu05200	Pathways in cancer	55	1.8512	0.040484
mmu05146	Amoebiasis	19	0.8384	0.041384
mmu03460	Fanconi anemia pathway	11	0.3702	0.042946
mmu03320	PPAR signaling pathway	15	0.5048	0.045273
mmu00592	alpha-Linolenic acid metabolism	7	0.2356	0.045563
mmu04080	Neuroactive ligand-receptor interaction	41	1.3800	0.047377
mmu04723	Retrograde endocannabinoid signaling	18	0.6058	0.048097
mmu05330	Allograft rejection	11	0.4854	0.048416
mmu05160	Hepatitis C	21	0.9267	0.049448
mmu04921	Oxytocin signaling pathway	25	0.8414	0.051647

Notes: ‘Count’: the number of genes in each term that are differentially expressed in the BD neurons.

Table S2. KEGG pathways showing bi-directional differential expression in the hippocampus of mice with acute/chronic mania or depression.

S3.1. KEGG pathways differentially expressed in the hippocampus of manic mice.

KEGG ID	Term	Count	%	p value
mmu04020	Calcium signaling pathway	40	1.3436	0.0014
mmu04921	Oxytocin signaling pathway	36	1.2092	0.0016
mmu04010	MAPK signaling pathway	51	1.7131	0.0027
mmu05166	HTLV-I infection	55	1.8474	0.0028
mmu05202	Transcriptional misregulation in cancer	36	1.2092	0.0039
mmu05150	Staphylococcus aureus infection	15	0.5038	0.0049
mmu04380	Osteoclast differentiation	28	0.9405	0.0085
mmu05134	Legionellosis	15	0.5038	0.0165
mmu05142	Chagas disease (American trypanosomiasis)	23	0.7725	0.0171
mmu05140	Leishmaniasis	16	0.5374	0.0203
mmu05218	Melanoma	17	0.5710	0.0241
mmu05200	Pathways in cancer	68	2.2841	0.0262
mmu05161	Hepatitis B	29	0.9741	0.0312
mmu05322	Systemic lupus erythematosus	29	0.9741	0.0338
mmu05133	Pertussis	17	0.5710	0.0345
mmu05146	Amoebiasis	24	0.8061	0.0371
mmu04060	Cytokine-cytokine receptor interaction	44	1.4779	0.0378
mmu04916	Melanogenesis	21	0.7054	0.0385
mmu04144	Endocytosis	48	1.6123	0.0543
mmu04014	Ras signaling pathway	40	1.3436	0.0680
mmu05205	Proteoglycans in cancer	36	1.2092	0.0699
mmu04725	Cholinergic synapse	22	0.7389	0.0745
mmu05203	Viral carcinogenesis	40	1.3436	0.0756
mmu00051	Fructose and mannose metabolism	9	0.3023	0.0770
mmu04612	Antigen processing and presentation	17	0.5710	0.0775
mmu00511	Other glycan degradation	6	0.2015	0.0832
mmu04151	PI3K-Akt signaling pathway	57	1.9146	0.0925
mmu04064	NF-kappa B signaling pathway	19	0.6382	0.0945
mmu05034	Alcoholism	35	1.1756	0.0951
mmu01100	Metabolic pathways	209	7.0204	0.0008

S3.2. KEGG pathways differentially expressed in the hippocampus of depressive mice.

KEGG ID	Term	Count	%	p value
mmu04020	Calcium signaling pathway	36	1.2195	0.0015
mmu04921	Oxytocin signaling pathway	32	1.0840	0.0024
mmu04010	MAPK signaling pathway	48	1.6260	0.0007
mmu05166	HTLV-I infection	49	1.6598	0.0033
mmu05202	Transcriptional misregulation in cancer	28	0.9485	0.0470
mmu05150	Staphylococcus aureus infection	13	0.4403	0.0110
mmu04380	Osteoclast differentiation	26	0.8807	0.0053
mmu05134	Legionellosis	12	0.4065	0.0637
mmu05142	Chagas disease (American trypanosomiasis)	6	1.1299	0.0735

mmu05140	Leishmaniasis	13	0.4403	0.0654
mmu05218	Melanoma	14	0.4742	0.0665
mmu05200	Pathways in cancer	15	2.8248	0.0503
mmu05161	Hepatitis B	9	1.6949	0.0141
mmu05322	Systemic lupus erythematosus	32	1.0840	0.0007
mmu05133	Pertussis	15	0.5081	0.0461
mmu05146	Amoebiasis	8	1.5065	0.0136
mmu04060	Cytokine-cytokine receptor interaction	39	1.3211	0.0414
mmu04916	Melanogenesis	21	0.7113	0.0098
mmu04144	Endocytosis	43	1.4566	0.0481
mmu04014	Ras signaling pathway	37	1.2533	0.0389
mmu05205	Proteoglycans in cancer	10	1.8832	0.0328
mmu04725	Cholinergic synapse	21	0.7113	0.0377
mmu05203	Viral carcinogenesis	40	1.3550	0.0112
mmu00051	Fructose and mannose metabolism	8	0.2710	0.0950
mmu04612	Antigen processing and presentation	15	0.5081	0.0935
mmu00511	Other glycan degradation	7	0.2371	0.0135
mmu04151	PI3K-Akt signaling pathway	58	1.9647	0.0059
mmu04064	NF-kappa B signaling pathway	8	1.5065	0.0050
mmu05034	Alcoholism	36	1.2195	0.0107
mmu01100	Metabolic pathways	198	6.7073	7.38E-06

Notes: ‘Count’: the number of genes in each term that are differentially expressed in the BD neurons.

Table S3. Clinical Characteristics of BD Subjects.

Subject ID	Gender	Age	Family History	Suicide ideation	Onset	Age of 1st E	Episode number	Diagnosis	Course of illness (month)	Alcohol history	Smoking history
BDI#01	M	65	N	Unk	Unk	Unk	Unk	BDI	Unk	Unk	Unk
BDI#02	M	59	Y	Unk	Unk	Unk	Unk	BDI, PTSD	Unk	Unk	Unk
BDI#03	M	57	Y	Unk	Unk	Unk	Unk	BDI. PTSD, ADHD	Unk	Unk	Unk
BDI#04	M	69	N	Unk	Unk	Unk	Unk	BDI	Unk	Unk	Unk
BDI#05	M	54	Y	Unk	Unk	Unk	Unk	BDI, PTSD	Unk	Unk	Unk
BDI#06	M	22	Y	Unk	Unk	Unk	Unk	BDI, PTSD	Unk	Unk	Unk

Note: M = Male; F = Female; C = Cell line; R= Recurrence; 1st E= First episode; BD I = Bipolar I Disorder; Y=Yes; N=No; Unk = Unknown, there was insufficient information to determine these phenotypes.

Table S4. Sequence of primers used for qRT-PCR analysis.

Gene	Sequence (5' to 3')
Syt7 (mouse)	Forward: 5’-GCTGCTCTTGTCCCTCTGCTAC-3’
	Reverse: 5’-CATGGCTTTGAGGTTTCGAGCTTT-3’
Syt7 (human)	Forward: 5’-AAGCGGGTGGAGAAGAAGAA-3’
	Reverse: 5’-CGAAGGCGAAGGACTCATTG-3’
GAPDH (mouse)	Forward: 5’-ATGACTCCACTCACGGCAAA-3’
	Reverse: 5’-TAGACTCCACGACATACTCAGC-3’
GAPDH (human)	Forward: 5’- TGTTGCCATCAATGACCCCTT-3’
	Reverse: 5’- CTCCACGACGTACTCAGCG-3’
CLOCK (human)	Forward: 5’- CTAGATCACAGGGCACCACC-3’
	Reverse: 5’- CCAAATCCACTGTTGCCCCT-3’
BMAL1 (human)	Forward: 5’- ACGGGGAAATCAGGGTGAAA-3’
	Reverse: 5’- TCTCGTCTGTAAAACTTGCCT-3’
Cry1 (human)	Forward: 5’- AAGAAAAGCTTGGGTGGCAAA-3’
	Reverse: 5’- TAAAGGGAAAGGGGAGGGGAA-3’
Cry2 (human)	Forward: 5’- GGAGCCTAAGACAGCACACC-3’
	Reverse: 5’- CACTGCAACCTGGTCTCCTC-3’
Npas2 (human)	Forward: 5’- AGAGGCAGCTTGAACCCAAA-3’
	Reverse: 5’- ACGGTGGCAATGAAGCAAAC-3’
Per2 (human)	Forward: 5’- TCCAGTCCCACTGCCATTAC-3’
	Reverse: 5’- GGTTTGACCCGCTTGGACTT-3’
CLOCK (mouse)	Forward: 5’- GGCGGTAGCGGTGAATTTTG-3’
	Reverse: 5’- GTGCTTTTCTTTCACGTGCCT-3’
BMAL1 (mouse)	Forward: 5’- GGCGTCGGGACAAAATGAAC-3’
	Reverse: 5’- TGCTTCTGTGTATGGGTTGGT-3’
Cry1 (mouse)	Forward: 5’- GGAAAGAAAGGCCTGGGTGG-3’
	Reverse: 5’- AAGAGAAAGGGGAGGGGAAC-3’
Cry2 (mouse)	Forward: 5’- GCAGAGAAGCCAATCACCAAC-3’
	Reverse: 5’- AAGCAGCCCTGGTAAGAAGA-3’
Npas2 (mouse)	Forward: 5’- TCCCACAAGTCCTCACACAC-3’
	Reverse: 5’- TGGTAACTCCTGGGGTAGGG-3’
Per2 (mouse)	Forward: 5’- TTGGGAGGCACAAAGTCAGG-3’
	Reverse: 5’- TTGCTGTCGCTGGATGATGT-3’
